# Detection of Developmental Asexual Stage-Specific RNA Editing Events in *Plasmodium falciparum* 3D7 Malaria Parasite

**DOI:** 10.3390/microorganisms12010137

**Published:** 2024-01-10

**Authors:** Md Thoufic Anam Azad, Tatsuki Sugi, Umme Qulsum, Kentaro Kato

**Affiliations:** 1 Laboratory of Sustainable Animal Environment, Graduate School of Agricultural Science, Tohoku University, 232-3 Yomogida, Naruko-onsen, Osaki, Miyagi 989-6711, Japan; 2 Department of Veterinary and Animal Sciences, University of Rajshahi, Rajshahi 6205, Bangladesh; 3 Division of Collaboration and Education, International Institute for Zoonosis Control, Hokkaido University, Nishi10-Kita 20, Sapporo 001-0020, Japan; 4 Department of Botany, Faculty of Biological Sciences, University of Rajshahi, Rajshahi 6205, Bangladesh

**Keywords:** gene modification, hemoglobin binding, mutation, time-course, variation

## Abstract

Transcriptional variation has been studied but post-transcriptional modification due to RNA editing has not been investigated in *Plasmodium*. We investigated developmental stage-specific RNA editing in selected genes in *Plasmodium falciparum* 3D7. We detected extensive amination- and deamination-type RNA editing at 8, 16, 24, 32, 40, and 46 h in tightly synchronized *Plasmodium*. Most of the editing events were observed in 8 and 16 h ring-stage parasites. Extensive A-to-G deamination-type editing was detected more during the 16 h ring stage (25%) than the 8 h ring stage (20%). Extensive U-to-C amination-type editing was detected more during the 16 h ring stage (31%) than the 8 h ring stage (22%). In 28S, rRNA editing converted the loop structure to the stem structure. The hemoglobin binding activity of PF3D7_0216900 was also altered due to RNA editing. Among the expressed 28S rRNA genes, PF3D7_0532000 and PF3D7_0726000 expression was higher. Increased amounts of the transcripts of these two genes were found, particularly PF3D7_0726000 in the ring stage and PF3D7_0532000 in the trophozoite and schizont stages. Adenosine deaminase (ADA) expression did not correlate with the editing level. This first experimental report of RNA editing will help to identify the editing machinery that might be useful for antimalarial drug discovery and malaria control.

## 1. Introduction

Malaria continues to be a global health threat [1]. Four species, *P. falciparum*, *P. vivax*, *P. ovale*, and *P. malariae*, cause human infection, with *P. falciparum* causing the most severe disease [2]. There is no fully effective vaccine against this pathogen, and it is acquiring resistance to first-line antimalarial drugs [3]. Wide evolutionary adaptation of *Plasmodium* spp. and lack of implementation of proper control strategies have allowed this pathogen to persist in endemic areas. Genetic variation is the essential phenomenon in parasites that allows them to adapt to the varied environments of their hosts and vectors, with single nucleotide polymorphism (SNP) being the most common type of genetic variation among the individual organisms. However, the adoption of beneficial SNP variations in the genome of an organism can be a difficult process. In contrast, transcriptional variation is a comparatively easier genetic change and adaptive mechanism in *Plasmodium* [4,5] and other organisms [6,7]. Transcriptional variation, including single-nucleotide variation (SNV), occurs at a much higher frequency in *Plasmodium* than in bacteria [4,5]. SNV can occur due to a transcriptional error or RNA editing [5].

RNA editing is a post-transcriptional mechanism that can alter nucleotides and thus recode the translation. It is caused by the deamination, amination, addition, or deletion of nucleotides by enzymes from the host and the organism itself. The most common deaminase enzymes are responsible for A-to-I RNA editing; C-to-U RNA editing is largely mediated by the APOBEC family of deaminases in humans [8,9]. The APOBEC enzymes have a considerable level of similarity in the groove they form for their substrate [10]. Over the last two decades, evidence has emerged demonstrating RNA editing in human ion channels and neurotransmitters [11,12,13]. In recent years, researchers have also explored RNA editing in nuclear genes and investigated RNA editing factors in plants. We previously revealed that *Arabidopsis thaliana* plant genes undergo RNA editing during the early developmental stages. RNA editing occurs in *Arabidopsis thaliana* at 12-day intervals, up to 20 percent of the specific transcript [14,15]. Our previous findings on RNA editing later expanded to greater aspect via deep sequencing and validation at greater scale [14,15]. Recently, plant RNA editing factors have been engineered to function in a human cell line, with high editing efficiency [16]. In the polar octopus K+ channel, editing is associated with external environmental pressure, specifically temperature adaptation [6]. In cephalopods, massive gene transcripts undergo RNA editing [7].

*Plasmodium* spp. have a wide variety of adenosine deaminases (ADAs) that convert adenosine into inosine; the inosine then functions as guanosine during translation [2] and has different ligand binding affinity. The *Pf*ADA-Asp176 residue is essential for the conversion of adenosine and especially for 5′-methylthioadenosine (MTA) [17]. ADAs are essential for *Plasmodium* survival [2]; therefore, we can predict that the RNA editing mechanism is also an essential adaptive mechanism for this parasite.

Multidrug resistance capacity, an evolutionary mechanism of the organism, is largely related to its SNV-like genome plasticity [3,18]. Genomic and post-transcriptional variations occur with high frequency in *Plasmodium* [5]. Transcriptional variation has been studied more extensively in recent years, thanks to advances in next-generation sequencing technologies. Large-scale data analyses now facilitate a better understanding of adaptivity and variation [4,5]. But we still have limited knowledge of the extension and causal factors of post-transcriptional variation, that is, RNA editing. tRNA editing has been reported in the *Plasmodium* apicoplast [19]. A complex mechanism of RNA editing has been observed in the mitochondria of trypanosomes [20]. RNA editing may alter the surface ligands of the parasites by altering single nucleotides. Such alterations may also affect the virulence capacity of the parasites. Studies have shown that certain single nucleotide alterations affect the sickle hemoglobin dependence of the parasites [21]. In *Plasmodium,* RNA modification (SNV) is observed within its 48 h life cycle at higher levels than bacteria [5]. But post-transcriptional modification due to RNA editing has not been fully investigated. Malarial parasites might have a stronger and more diversified RNA editing machinery than humans and plants. As *Plasmodium* adapts to its host and vector with changes in temperature, single nucleotide alterations by RNA editing may serve as an adaptive switch for *Plasmodium* spp. Gene duplication and genome complexity with higher AT-rich regions make it difficult to find accurate RNA editing; therefore, we (a) screened the literature on SNV, i.e., selected the predictable variation sites [5], (b) detected RNA editing in the highly synchronized parasite mRNAs with Sanger sequencing, (c) removed the detected sites due to multiple-copy variation of a gene, and (d) analyzed NGS sequencing and data on an integrative genomics viewer (IGV) to confirm RNA editing sites.

A better understanding of the mechanisms and factors involved in RNA editing will improve control strategies and drug development against malaria. In this study, we examined RNA editing at 8 h intervals at three developmental stages: ring, trophozoite, and schizont. We found that *Plasmodium* nuclear transcripts undergo RNA editing, exhibiting a stage-specific pattern. We also quantified the RNA editing level. We applied detailed approaches to detect developmental stage-specific RNA editing events in selected genes of *Plasmodium* and their effect on the RNA’s secondary structure, protein structure, and functionality.

## 2. Materials and Methods

### 2.1. Parasite Culture Conditions and Synchronization

*Plasmodium falciparum* 3D7 parasites were cultured in 10 mL flasks containing RPMI 1640, 25 mM HEPES, 100 μM hypoxanthine, 12.5 μg/mL gentamicin sulfate, and 62.5 μg/mL NaHCO_3_, as previously described [22,23]. Cell cycles were observed for one week, and the early ring-stage parasites were treated with filter-sterilized 5% sorbitol for initial synchronization. Sorbitol-treated parasites were cultured for another week to increase the number of synchronized cell cycles, and the media was changed every 24 h. The culture was diluted to maintain about 5% parasitemia at 3% hematocrit. When the parasite concentration reached the desired concentration and the late-stage schizonts were ready to release merozoites, the mature schizonts were collected from the trace amount of the developmental stage using the MT biotech MACS, MS column purification system, and Percoll–sorbitol synchronization. The schizonts were allowed to invade the RBCs for 5 h, and then a 5% sorbitol treatment was conducted to destroy the late-stage and remaining schizonts. At 8 h intervals, 6 stage samples (8, 16, 24, 32, 40, and 46 h) were collected for RNA extraction. Note that the sixth stage sample was collected at 46 h instead of 48 h.

### 2.2. Extraction of RNA and cDNA Synthesis

Total RNA was extracted from the six stage samples using Trizol reagents (Invitrogen) according to the manufacturer’s instructions and was treated with RNase-free DNase to remove genomic DNA contamination. The RNA samples were then purified using phenol–chloroform extraction and ethanol precipitation if needed, and were then quantified using a NanoDrop spectrophotometer (Thermo Scientific, Waltham, MA, USA). Purified RNA was subjected to cDNA synthesis using reverse transcriptase (Superscript III, Invitrogen, Waltham, MA, USA), oligo dT primers, and random hexamer (Superscript III, Invitrogen, Waltham, MA, USA).

### 2.3. Selection of Genes for the RNA Editing Study

The studied genes were selected based on a previous study [5] with high transcriptome level variation. Control raw data were downloaded and searched for highly variable transcriptional-level regions of the 28S rRNA genes PF3D7_0112700, PF3D7_0532000, PF3D7_0726000, PF3D7_1148640, and PF3D7_1371300. Other selected genes were PF3D7_0216900, protein of unknown function; PF3D7_0501200, parasite-infected erythrocyte surface protein; PF3D7_1227200, potassium channel K1; and PF3D7_1149000, antigen 332/DBL-like protein (Supplementary Table S1). To identify the best candidate genes involved in RNA editing, the full-length genomic DNA, mRNA, and cDNA sequences of each gene were obtained from Plasmo DB (https://plasmodb.org/plasmo/app (accessed on 26 October 2023)).

### 2.4. Primer Design

Primers were designed using Primer3 (bioinfo.ut.ee/primer3-0.4.0/primer3/ (accessed on 26 October 2023)) and verified using the NCBI Primer-BLAST tool. In the case of failure or inappropriate outcome with the first set of primers, new primer sets were designed. Primers were purchased from Eurofins (Yokohama, Japan) in TE buffer at a concentration of 50 pmol/μL in a salt-free condition. Each primer was diluted to a working concentration of 10 pmol/μL in TE buffer.

### 2.5. Sequencing of PCR Products

To identify RNA editing sites, 1% agarose gel purified PCR products were subjected to direct sequencing on a genetic analyzer using the BigDye Terminator v3.1 Cycle Sequencing Kit (Applied Biosystems, Austin, TX, USA). PCR products were purified using the QIAquick gel extraction kit, and concentration was measured using the NanoDrop. Sequencing was performed by the Macrogen company, Kyoto, Japan. Sequences of the reverse strands were reverse complemented using online software (https://www.bioinformatics.org/sms/rev_comp.html (accessed on 26 October 2023)). The raw sequencing data were analyzed using Sequence Scanner software version 2 (Applied Biosystems) and 4Peaks software, v1.8. All sequences were aligned with the *Plasmodium falciparum* genome sequence using BLAST and Plasmo DB (https://plasmodb.org/plasmo/app (accessed on 26 October 2023)).

### 2.6. Quantification of Different RNA Editing Events

RNA editing events were quantified from the Sanger sequencing results using the peak height ratio method. The peak heights of the corresponding dual peaks were calculated using 4Peaks software. RNA editing (%) was quantified based on the maximum peak height, according to the following equation [24]: RNA editing (%) = [C height/(T height + C height)] × 100.

### 2.7. Prediction of mRNA and Protein Structures

The secondary structures of mRNAs were predicted using the RNA fold web server http://rna.tbi.univie.ac.at/cgi-bin/RNAWebSuite/RNAfold.cgi (accessed on 26 October 2023), with default parameters. The I-TASER server was used to assess secondary protein structure and ligand binding activity [25,26]. AlphaFold [27,28], a protein structure prediction database, was also used. AlphaFold produces a per-residue confidence score (pLDDT) between 0 and 100. Some regions below 50 pLDDT may be unstructured in isolation.

### 2.8. Tight Synchronization and RNA Extraction for NGS

Parasites were synchronized using the 5% sorbitol and Percoll–sorbitol techniques. After Percoll–sorbitol synchronization, the parasites were allowed to invade fresh RBCs for 5 h to obtain fresh ring-stage parasites. After a 5 h incubation, the parasites were again synchronized by use of 5% sorbitol to kill the remaining mature-stage parasites. RNA was extracted at 16 h, 24 h, 32 h, and 40 h to obtain RNA from the ring, trophozoite, and schizont stages. RNA quality was assessed using a NanoDrop spectrophotometer (Thermo Fisher Scientific, Waltham, MA, USA), and the RNA was stored at −80 °C until use.

### 2.9. RNA Integrity Test to Check for Hidden Breaks (HBs)

The total RNA integrity of the 16, 24, 32, and 40 h samples was tested using an Agilent Technologies 2100 Bioanalyzer (or 2200 TapeStation) with an RNA integrity number (RIN). This service was provided by Macrogen, Japan. Values greater than or equal to 7 indicated good-quality RNA. The presence of a clear 28S rRNA peak was considered to indicate no HBs.

### 2.10. Library Preparation for Total mRNA Sequencing

Samples were sent to Macrogen, Japan for library preparation and total mRNA NGS sequencing. Sample initial quality control was also confirmed by Macrogen. A TruSeq stranded mRNA library preparation kit was used. Samples were sequenced by paired ends at a 101 bp read length using the NovaSeq sequencing platform. After sequencing, the results were checked and the quality was again assessed by Macrogen. Approximately >4 Gbp data for each sample with GC content > 30% and Q30 > 94% reads were obtained for each sample; md5 values were checked for data integrity when downloaded.

#### 2.10.1. Quality Control

After sequencing, initial quality control was performed by Macrogen. We checked the quality using FastQC and FastTP. Only good-quality reads were considered for next-step analysis. Q20 reads were considered for next-step analysis. Trimgallor was used for trimming and removing low-quality reads.

#### 2.10.2. Data Analyses

STAR alignments were used to align the paired-end clean reads with the reference genome of *Plasmodium falciparum*. BAM files were produced and Samtools was used to obtain BAM index files for further analysis.

### 2.11. RNA Seq Data Analysis and Integrated Genomics Viewer (IGV) Analysis for RNA Editing Event Detection and Quantification

A downloaded version of IGV (version 2.16.01) for Mac was used to view the STAR-aligned BAM files. The BAM files of the representative samples with three replicates were loaded, and randomly selected targeted genes’ loci were examined in IGV to check the RNA editing. Three replicates for each stage-dependent time were loaded in IGV, and the targeted nucleotides/loci were examined for editing changes.

### 2.12. Stage-Specific Expression of RNA Editing-Related Genes

Raw reads were further analyzed using iDEP [29]. Raw read counts were uploaded in iDEP, and the reference genome of *Plasmodium falciparum* was selected or auto-matched to the best match to the *Plasmodium falciparum* genome. DSEq1 and Dseq2 were performed with iDEP. Raw read data were further analyzed using iDEP v1.0 [29]. The data were first filtered to remove reads below 0.5 CPM in at least 1 sample(s). Then, the data were transformed with EdgeR using a pseudocount of 4. Missing values were imputed using the gene median. Of the 5767 genes in the 12 samples (three replicates for each), 5397 passed the filter. These 5397 genes were converted to Ensembl gene IDs. Transformed group expression was analyzed with iDEP 1.1 [29]. Three biological replicates for each condition were analyzed.

## 3. Results

### 3.1. RNA Editing Event Detection

**Figure 1:**
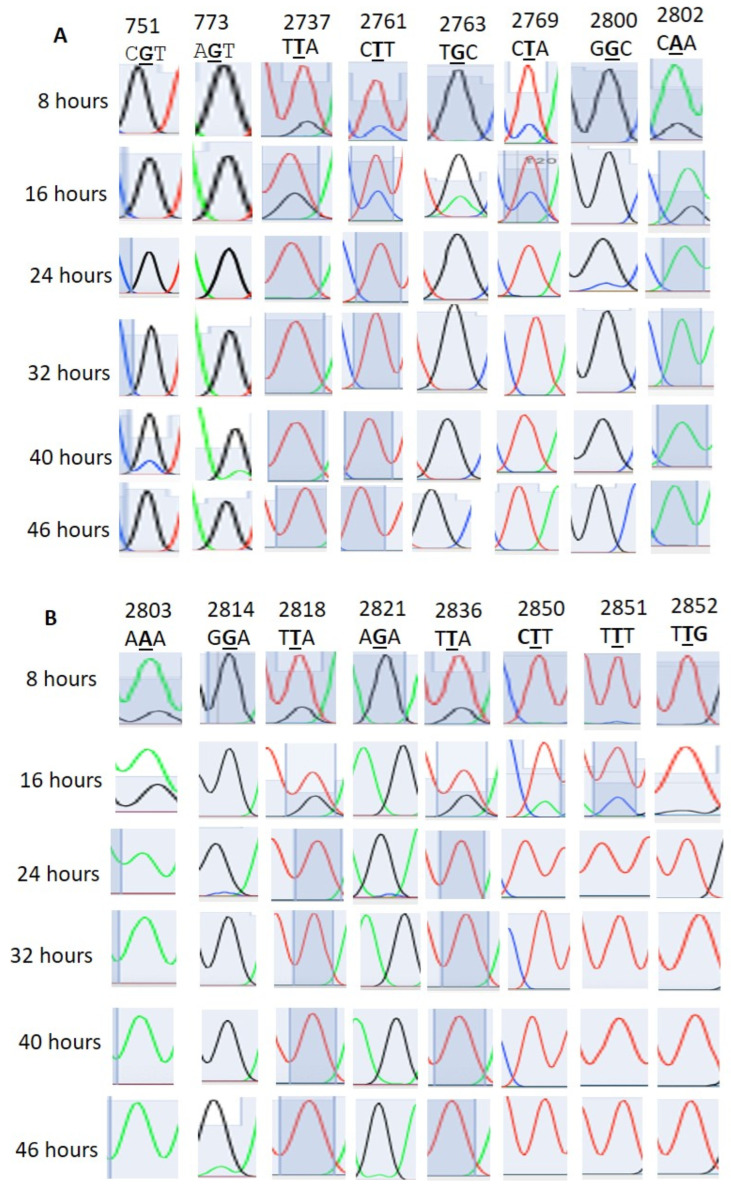
(**A**,**B**) Stage-specific RNA editing events in 28S rRNA.

**Figure 2:**
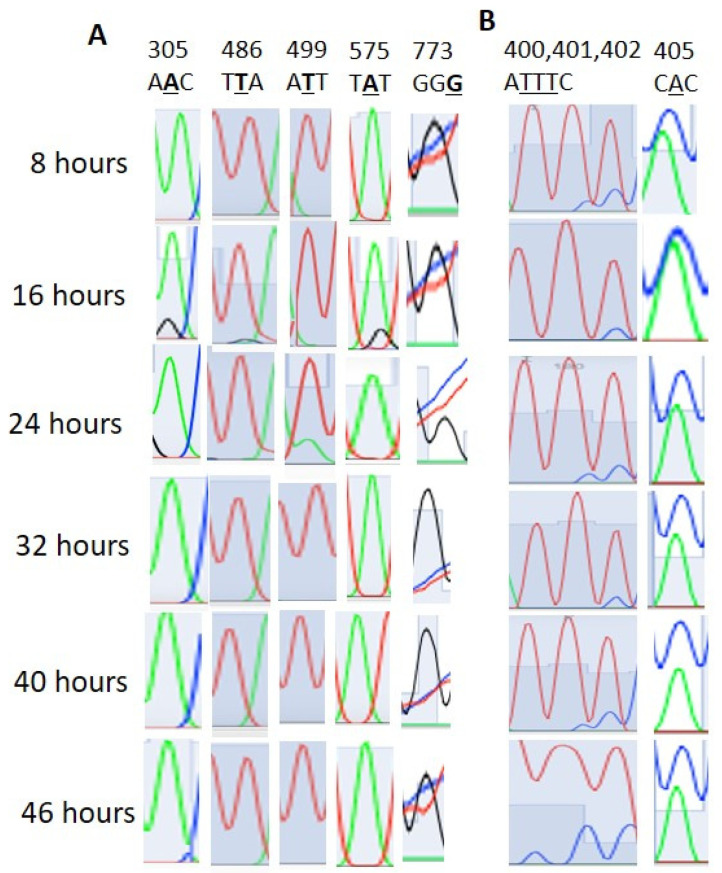
(**A**). Stage-specific RNA editing event positions in the PF3D7_0216900 gene-conserved protein, unknown function, of *Plasmodium falciparum*. (**B**). Stage-specific RNA editing event positions in the PF3D7_0501200/PIEPSP2 gene of *Plasmodium falciparum*. AATTTAAATTTCCAC> AAAATTTCCCCCCC, UTR region upstream.

We found that 28S rRNA genes with considerable homology were present in chromosomes 1, 5, 7, 11, and 13 (Supplementary File 1). The editing events in the homologous regions were considered to be real editing events. Here, the 28S rRNA gene PF3D7_0112700 was considered the standard for the RNA editing locus and site specificity. In Table 1, apart from events 2633, 2645, and 2669, all the other events were confirmed as RNA editing (Figure 1). The 2633, 2645, and 2669 events were due to multiple copies of the 28S rRNA genes (Supplementary Figures S1–S3). Both amination (T>C, G>A) and deamination (A>I, C>T) types of editing were observed. T>G, T>C, G>A, and T>C editing occurred with 28%, 31%, 24%, and 31% of the transcripts of the ring-stage parasites at 16 h, respectively (Table 1 and Figure 1). At 8 h, T>G, T>C, and T>C editing occurred at 17%, 20%, and 22%, respectively, in sites 2737, 2761, and 2769. G>A editing was absent at 8 h. In addition to sites 2633, 2645, and 2449, other sites also showed RNA transcript/copy number variation (Supplementary Figures S1–S3). The PF3D7_0216900 gene is present as a single copy in *Plasmodium falciparum*. Editing events were observed at sites 305, 499, 486, 575, and 773, with 305 A>G, 486 T>G, 499 T>A, 575 A>G, and 773 G>T (Figure 2A).

In the PF3D7_0501200/parasite-infected erythrocyte surface protein, editing was also observed (T>C and A>C) at sites 400, 401, 402, and 405 in the 5′ UTR region (Figure 2B). In PF3D7_1227200, editing was not detected at sites 1936 and 1938, which were previously predicted to undergo editing (Supplementary Figure S4). In PF3D7_1149000/antigen 332, a DBL-like protein, 4151st position A>G editing was not detected although it was previously predicted to undergo editing (Supplementary Figure S5). We also found no T>A editing at PF3D7_0815200/importin subunit beta position 2658 (Supplementary Figure S6). We checked gDNA sequences on a limited scale (Supplementary Figure S7). Although RNA editing signals were observed in the stage-specific mRNA sequencing data, no such peaks were observed in the gDNA sequence (Supplementary Figure S7).

### 3.2. RNA Integrity Test to Check for Hidden Breaks (HBs) 

Full or partial hidden breaks (HBs) [30] may be represented as false RNA editing. The RIN numbers of the 16, 24, 32, and 40 h RNA samples were tested by Macrogen, Japan. This test was conducted to check for HBs at any stage. The bioanalyzer result showed that HBs were not present in any stage-specific RNA (Supplementary Figures S7 and S8).

### 3.3. RNA Editing Affects RNA and Protein Secondary Structures

**Figure 3:**
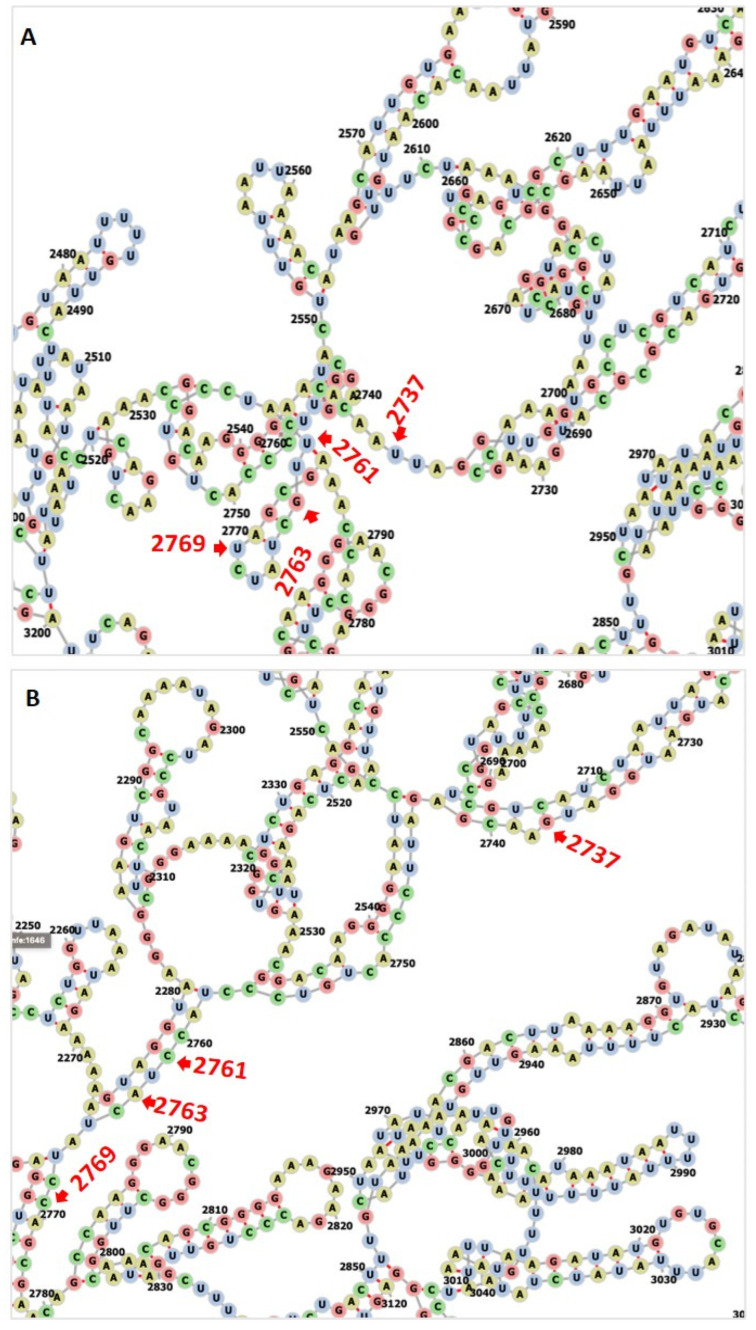
(**A**) The unedited transcript forms a more complex structure than the edited one. (**B**) RNA editing transforms most of the loop structure to a stem structure in PF3D7_0112700, 28S ribosomal RNA, ncRNA gene.

**Figure 4:**
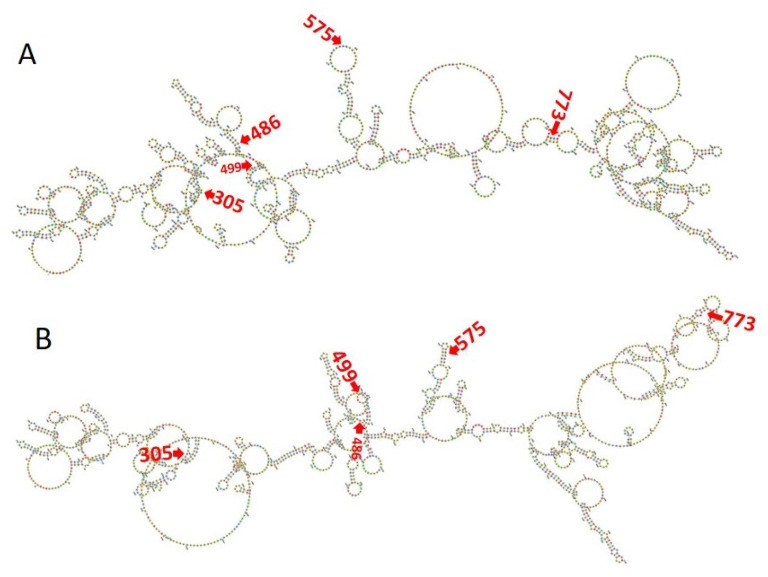
(**A**) The unedited transcript forms a more complex structure than the edited one. (**B**) RNA editing transforms most of the loop structure to a stem structure in PF3D7_0216900.

**Figure 5:**
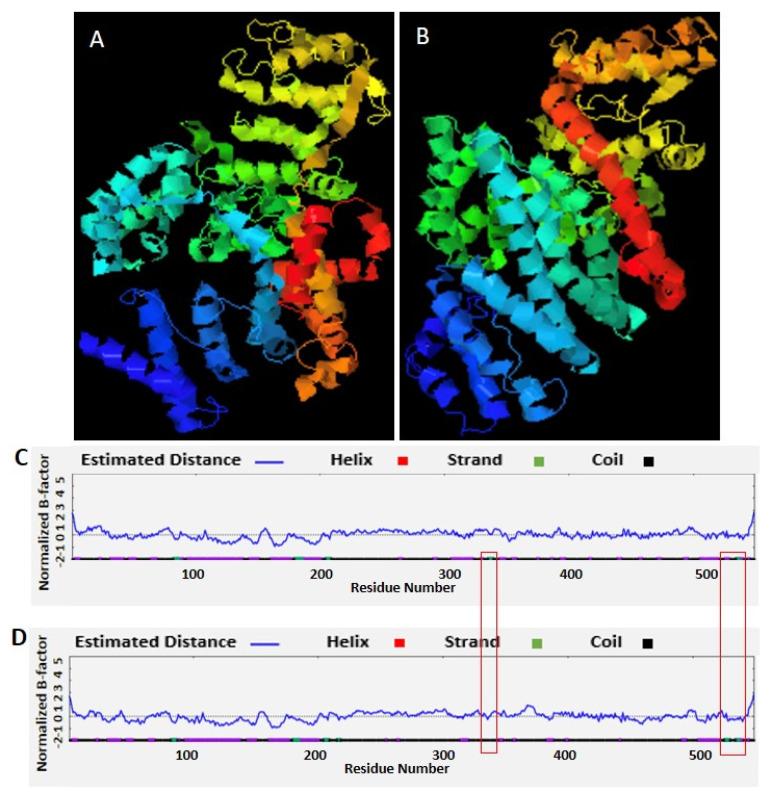
RNA editing in the PF3D7_0216900 gene of *Plasmodium falciparum* affects protein secondary structure. (**A**) Unedited protein secondary structure. (**B**) Edited protein secondary structure. (**C**) Unedited protein with five beta sheets. (**D**) Edited protein with six beta sheets.

**Figure 6:**
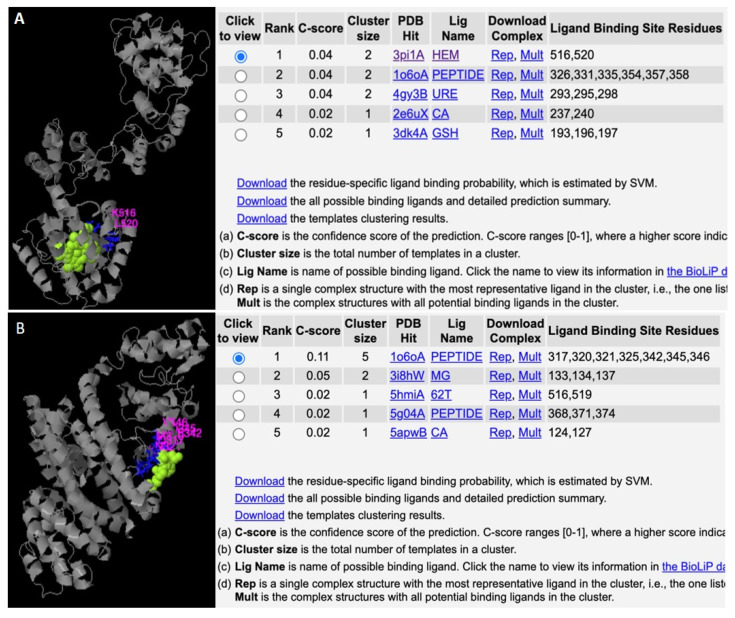
Altered ligand binding affinity due to RNA editing. (**A**) Unedited protein binds to hemoglobin, which is an important activity for parasite development. (**B**) Protein loses its hemoglobin-binding activity due to RNA editing.

In the 28S rRNA, RNA editing at nucleotides 2737, 2761, 2763, and 2769, i.e., T>G, T>C, G>A, and T>C, altered the RNA secondary structure (Figure 3, Supplementary Figure S10). In PF3D7_0216900, RNA editing affected positions 305 A>G, 486 T>G, 499 T>A, 575 A>G, and 773 G>T (Figure 4). Nucleotide 486 is in the stem of the RNA duplex, where, due to RNA editing, it helps to form a loop structure (Figure 4, Supplementary Figure S10). Protein secondary structure was also altered due to RNA editing. Residues altered in PF3D7_0216900 included asparagine/N > S/serine, isoleucine, I > methionine/Met, phenylalanine/F > I/isoleucine, and Y/tyrosine > C/cysteine (Supplementary File 2). The unedited protein contained five beta sheets, whereas the edited protein contained six beta sheets (Figure 5). We observed the ligand hemoglobin binding capacity of the unedited protein to the 516 and 520 residues, whereas that of the edited protein was lost as most of the priority ligand binding residues were changed at 317, 320, 321, 325, 342, 345, and 346 (Figure 6). According to AlphaFold, the pLDDT score for residue 516 was very high (pLDDT > 90) and that for residue 520 was even higher (90 > pLDDT > 90) (Supplementary Figure S12). I-TASSER GO information on the biological process with a higher GO score (GO:0009894) indicated that this protein was responsible for a catabolic process (Supplementary Figure S13.)

### 3.4. RNA Seq Data Analysis and IGV Analysis for RNA Editing Event Detection

NGS of the 16 h, 24 h, 32 h, and 40 h mRNA samples was performed by Macrogen, Japan with the NovaSeq sequencing platform. The BAM files for the three replicates of each sample were loaded in IGV, and the best-match genome file (*Plasmodium falciparum* 3D7) was compared. The specific chromosome and gene identification numbers were used to navigate the desired read and nucleotide location. The sites where there was nucleotide alteration in most of the samples compared with the genomic sequence were considered the RNA editing sites. Randomly selected positions—2737 T>G, 2761 T>C, 2763 G>A, and 2769 T>C—were confirmed as editing events, along with their respective editing percentages (Supplementary Figures S14–S17). Both the RNA editing quantifications from Sanger sequencing (Table 1) and the IGV detection (Table 2) indicate that RNA editing occurred more frequently in the ring stage (8–24 h) than in the trophozoite (32 h) and schizont stages (40 h, 46 h).

### 3.5. Stage-Specific Expression of RNA Editing-Related Genes

**Figure 7:**
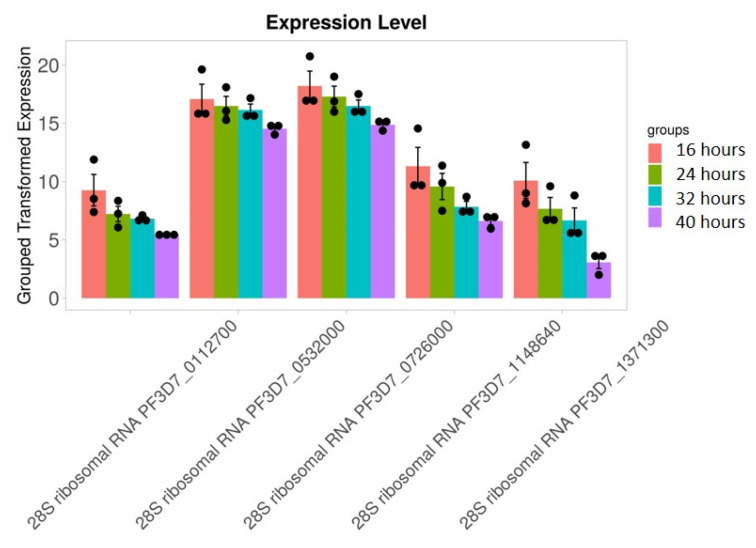
28S rRNA genes expression is higher in early-stage malarial parasites. Greater RNA modification, including RNA editing, was observed during the early stages. RNA modification is an important phenomenon in this parasite. Black dots with error bars indicate standard error.

**Figure 8:**
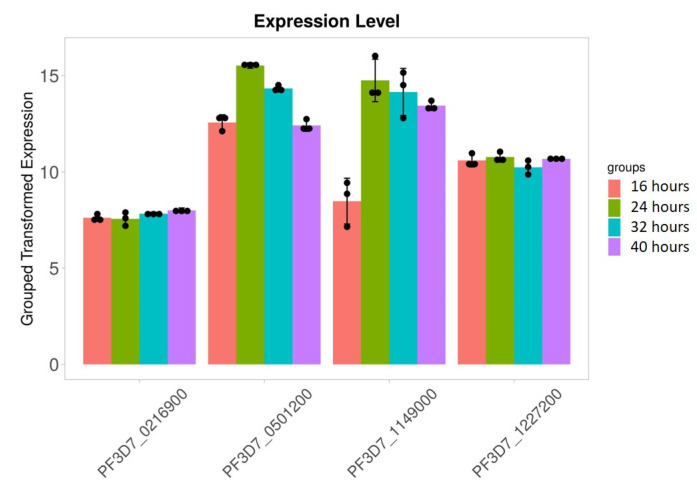
Stage-specific expression of the RNA editing in studied genes. Black dots with error bars indicate standard error.

**Figure 9:**
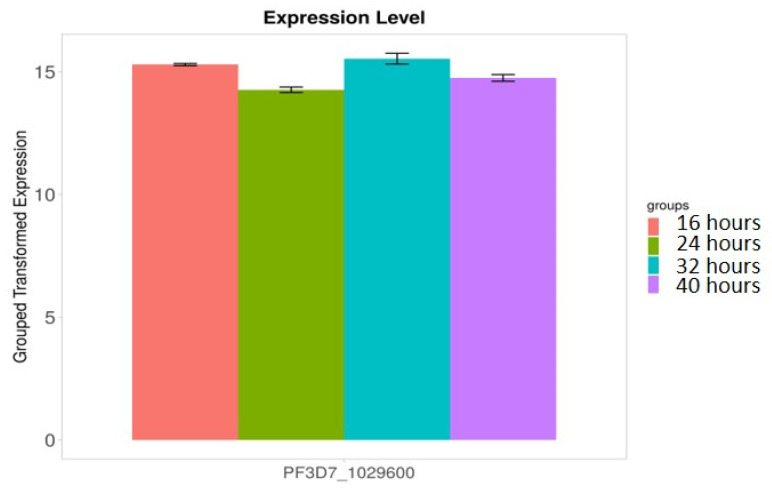
ADA expression does not change in a stage-specific manner. Error bars indicate standard error. This gene is essential for parasite survival and adenosine modification. ADA is responsible for adenosine modification observed in vitro [17].

We found that 28S rRNA gene expression was higher in early-stage malarial parasites, except for PF3D7_0801100 (Figure 7). The higher expression in the early ring-stage parasites gradually decreased as the life cycle progressed to the 40 h schizont-stage parasites. Greater RNA modification, including RNA editing, was observed in the early stages. Among the expressed 28S rRNA genes, the expression of PF3D7_0532000 and PF3D7_0726000 was higher (Figure 7). The transcripts of these two genes, along with others, were found in higher amounts during the ring stage compared to the trophozoite and schizont stages (Figure 7). PF3D7_0216900/protein of unknown function; PF3D7_0501200/parasite-infected erythrocyte surface protein; PF3D7_1227200/potassium channel K1; and PF3D7_1149000/antigen 332, DBL-like protein expression, was similar across almost all developmental stages (Figure 8). ADA expression did not change in a stage-specific manner (Figure 9).

## 4. Discussion

RNA editing is one of the important post-transcriptional modifications that contributes to RNA diversity, thereby influencing transcript diversity and protein functionality. Ribosomes play a vital role in molecular and cellular functions. The synthesis of the ribosome is a tightly programmed cellular process and inevitably linked to cell growth and multiplication [31]. Different types of SNV, like A>T, T>A, A>G, G>A, A>C, C>A, T>C, C>T, T>G, G>T, C>G, and G>C have been reported in *Plasmodium falciparum,* some of which may include RNA editing [5]. Among the SNVs, we confirmed that RNA editing events occur in a developmental stage-specific manner and observed the effect of RNA editing on 28S rRNA. Since 28S sRNA is much longer than other rRNA, it is more prone to mutation [32].

RNA editing occurs in the editing locus. The editing locus is mainly defined by the binding place and activity of the enzyme or enzyme complex that is responsible for RNA editing [33]. This indicates that both the deaminase and aminase types of enzyme systems may present in *Plasmodium* or that the deaminase enzymes may recognize and perform the amination process simultaneously. The substrate recognition groove for deaminase enzymes in *Plasmodium* is diversified [17]. *Plasmodium* adenosine deaminase (ADA) binds to not only adenosine (A) but also the noble binding pocket for inosine (I) [17] that acts as a homologue of guanosine (G), indicating that it may also be responsible for the amination reaction. We validated the RNA editing sites in the 28S rRNA genes and other single-copy genes in *Plasmodium.* On a limited scale, we checked the gDNA and found no editing signals in the Sanger sequencing peaks. Genome-wide detection and validation of RNA editing are needed in *Plasmodium,* with the available tools. In some regions, we found 100% variation that normally means base substitution. It has been reported that 100% editing is unrealistic [33]. So, these regions with 100% variation cannot be the result of RNA editing. They may be due to different isolates or other factors, such as different cultural conditions and sample preparation of the parasites. In one report [5], whole RNA was collected from the bulk culture to observe RNA variation based on a computational analysis, whereas we collected tightly synchronized parasites in a stage-specific manner for our RNA editing study. In the previous report, the A>G and T>C SNV comprised approximately 28% and 20%, respectively [5].

The 28S rRNA is cleaved into two almost equal halves (called an HB) in some eukaryotes [30]. A partial or complete HB in the middle of 28S rRNA may be misrepresented as RNA editing. We checked whether an HB [30] occurred in any stage of *Plasmodium* and found no HB or the presence of the 16s rRNA peaks using spectrophotometric analysis [30].

In this study, we observed that RNA editing occurred within a relatively short period of 8 h. In our previous study in HEK-293 cells, we observed that a considerable level of RNA editing occurs in engineered human deaminase enzymes within a short period [34,35,36,37,38]. Recently, it was reported that adenosine deaminase acting on RNA (ADAR) enzymes in birds evolved at 40 °C and targeted different groups of temperature-sensitive RNAs [39]. *Plasmodium* RNA recognition by ADAR complementary factors may be altered during the early ring and late schizont stages, when the organism faces higher core body temperature in its host. Temperature may also affect the expression of RNA editing factors and the structure of the RNA [40]. Therefore, RNA editing may play a vital role during the rapid and intermittent adaptation of malaria organisms to higher core body temperature.

The considerable level of similarity among the multiple copies of a gene [41,42] is a phenomenon in *Plasmodium* spp. In *Plasmodium,* the PF3D7_0112700, PF3D7_0532000, PF3D7_0726000, PF3D7_1148640, and PF3D7_1371300 genes are considered ncrRNA (plasmodb.org). Most of the transcriptional RNA consist of rRNA. Although these transcripts seem to have no protein-coding potentiality, they may regulate diverse molecular and ultimately biological functions [43]. Hence, any alterations in these transcripts may impact their functions and the adaptability of *Plasmodium*. Although it is reported that the different types of ribosome (A-type and O-type) are distinct to the different developmental stages, it also reported that they share some of the ribosomal RNA from the different stages [44]. We found that the stage-specific parasites not only utilize a distinct type of 28S rRNA in their blood-based developmental stages but also share the other variants of 28S rRNA.

RNA editing alters protein secondary structure. Several alpha helices and beta sheets were altered due to RNA editing. The number of beta sheets increased due to RNA editing. Variation in 5S, 5.8S, 18S, and 28S rRNA has been observed between individuals and within individuals [45]. Within individual nucleotide variation, expression at the tissue-specific level has been observed [45]. The heterogenicity of the 28S rRNA generates ribosomal code for the diversified mRNAs; thus, rRNA acts as a filter for the targeted mRNAs [31]. This implies that variations in 28S rRNA and protein translation may significantly influence the diversity of other proteins, emphasizing the vital role of these genes in parasite developmental stages. Modification and recoding by RNA editing of such transcripts may affect parasite development and virulence.

Most previous reports on RNA editing examined the expression of the RNA editing enzymes. The editing enzymes are primarily deaminase enzymes; however, in plants, a large group of PPR proteins also plays a role in RNA editing [46,47,48]. In animals and plants, deaminase gene expression is proportional to RNA editing [33]. The ADA gene is essential for adenosine modification in *Plasmodium* as well as parasite survival [2,17]. ADA is responsible for adenosine conversion in vitro [17]. Therefore, in malarial parasites, ADA and other groups of genes may be involved in RNA modification, RNA editing, and parasite adaptation. ADA in *Plasmodium falciparum* and *Plasmodium vivax* greatly differ in their substrate recognition. In *Plasmodium*, deaminase enzymes, PPR proteins, and cytidine deaminase are present and reported in a few studies. We found that RNA editing is mainly observed in the early parasitic developmental stages. The ADA factor is responsible for RNA editing but does not correlate with the expression of this gene. It likely relates to the protein expression of ADA and RNA editing. The assumption will be proven if the protein expression of ADA is detected and the correlation with RNA editing is found. Another possibility might be that there are complementary factors related to *Plasmodium* RNA editing that have not yet been reported. Other genes responsible for RNA modification may need to be identified. It was assumed that this organism goes through numerous transcriptional errors during its multiplication and adaptation to environments. However, the organism is well equipped to maintain its AT-rich genome (approximately 70%) with its transcriptional machinery [49].

RNA editing greatly affects RNA stability [50], secondary structure, and protein function [40]. In Plasmodium, apicoplast genes undergo RNA editing [19]. RNA editing of the rpl2 gene occurs in the 20 h ring-stage parasites [19]. Our study also observed that most of the nuclear gene RNA editing events occur during the ring stage of the parasites. Together, these findings suggest that the RNA editing machinery in *Plasmodium falciparum* is more functional in the early developmental stages.

In unedited peptides, hemoglobin binds to residues 516 and 520, which have high pLDDT values, according to AlphaFold [27,28]. Therefore, these two functional residues might be important for the function of this protein in hemoglobin internalization in malaria parasite metabolism in the initial stages of the life cycle. Hemoglobin is a vital nutrient and source for amino acids [51] for the invading malaria parasite during the asexual developmental stage [52]. Almost half of the hemoglobin contained in RBCs is consumed by the parasite, and the excess hemoglobin is converted to hemozoin to avoid toxicity [52]. Hemoglobin binding, internalization, and degradation is an important step in malaria drug development [52,53,54]. I-TASSER GO information (GO:0009894) indicated that this protein is also involved in a catabolic process. The PF3D7_0216900 gene may play a role in the breakdown of hemoglobin, serving as a nutrient source for *Plasmodium*. The protein therefore may be an important protein of interest for malaria parasites, and RNA editing may play a vital role favoring the parasite. Ring-stage parasites undergo profound morphological alteration, remodeling host RBCs to cope with cellular and host factors [51]. Binding to the hemoglobin likely starts at the early to middle stage of the ring stage, and therefore the RNA editing and the affinity for hemoglobin are reduced at the later stages. Signaling for early hemoglobin uptake may start at the early parasitic state to accelerate parasitic growth. Therefore, PF3D7_0216900 may play a vital role in the parasite’s survival adaptation and remodeling of the host erythrocytes [55] through hemoglobin metabolism.

It has been reported that, due to a single nucleotide change, *Plasmodium* spp. can acquire resistance to the available and newly developed antimalarial drugs [3]. Ducks’ and hummingbirds’ elevated core body temperature reprograms their deaminase systems. Then, the deaminase system recognizes the elevated temperature-sensitive RNA and thus they adapt to their environment [39]. Recently, it was reported that temperature influences RNA editing in the octopus neural proteome and affects the protein function to a considerable degree [40]. RNA editing might also be an important mechanism of adaptation in malarial pathogens [39,40,50,56,57]. Malaria organisms adapt to a wide range of hosts and to completely different environments. Therefore, RNA editing may play a vital role in adaptation, especially to the transient and intermittent febrile temperature of the host. Elucidation of such kinds of adoptive mechanisms might be an important tool for controlling this deadliest pathogen.

*Plasmodium* species were thought to contain many SNVs and transcriptional errors, but this organism can tightly control every step [58], including gene regulation of chromatin structure [59], transcription, and translation [60], which are essential for the adaptation and survival of the parasite in multiple hosts and environments. To validate the editing points, we tested randomly selected positions—2737 T>G, 2761 T>C, 2763 G>A, and 2769 T>C—using NGS-processed data and the IGV view. In most cases, Sanger sequencing was unable to detect low levels of editing. Conversely, the IGV view revealed low-level editing at 32 h trophozoite and 40 h schizont stages. Our IGV data revealed that RNA editing happens in the early stage of this parasite, and this is a biological phenomenon in *Plasmodium*. In addition, the tight stepwise [61] control of gene regulation in the parasite may support transcriptional diversity to some extent. Our study revealed that RNA editing contributes to transcriptional diversity in different developmental stages. The life cycle of *Plasmodium falciparum* is 48 h. Timing may be critical for the enzymatic activity of the different RNA modification machinery in the parasite genome and cytoplasm in a stage-specific manner. Recent reports indicate that RNA editing may occur within hours in octopus ganglionic cells, resulting in functional diversity in the motor protein kinesin and another essential protein, synaptotagmin [40]. Additionally, stage-specific stress-responsive RNA editing has been reported in fungi [62].

## 5. Conclusions

This is the first report of stage-specific RNA editing in the *Plasmodium* nuclear transcript. Confirming the presence of RNA editing events in the *Plasmodium* transcript establishes the foundation for studying the mechanisms and functions of these extensive post-transcriptional modifications. The probable factors related to RNA editing need to be investigated in *Plasmodium* spp. Genome-wide RNA editing events need to be investigated utilizing tools like REDItools, RES-scanner, JACUSA, etc. Multiple approaches should be considered for the precise detection of RNA editing in *Plasmodium*. Single-cell and long-read sequencing techniques, suitable for the higher AT-rich nucleotide content, along with appropriate validation techniques, are needed for the comprehensive detection of RNA editing in *Plasmodium* on a larger scale. Heat stress adaptation, multiplication, and understanding the factors related to RNA editing will be essential in controlling this dangerous drug-resistant pathogen.

## Figures and Tables

**Table 1:** Stage-specific transcriptional variation and RNA editing in the 28S ribosomal RNA gene. Variations were observed mostly in the 2633-to-2852 locus. With the exception of 2633 to 2649, all other variations were due to RNA editing.

Variation/Editing Site in Transcript; Gene ID PF3D7_0112700	Variation/Editing Types	Genomic Sequence	Stage-Specific Editing%					
			8 h	16 h	24 h	32 h	40 h	46 h
751	G>C	TCGTC	0%	0%	0%	0%	12%	0%
773	G>A	GAGTC	0%	0%	0%	0%	10%	0%
2633	A>C	AAATT	100%	100%	100%	100%	100%	100%
2645	T>C	TTTAA	100%	100%	100%	100%	100%	100%
2649	T>C	ATTAA	100%	100%	100%	100%	100%	100%
2649	C>G	ATCAA	20%	35%	0%	0%	0%	0%
2737	T>G	ATTAA	17%	28%	0%	0%	0%	0%
2761	T>C	ACTTG	20%	31%	0%	0%	0%	0%
2763	G>A	TTGCT	0%	24%	0%	0%	0%	0%
2769	T>C	TCTAG	22%	31%	0%	0%	0%	0%
2800	G>C	TGGCA	0%	0%	16%	0%	0%	0%
2802	A>G	GCAAA	20%	25%	0%	0%	0%	0%
2803	A>G	CAAAA	17%	27%	0%	0%	0%	0%
2814	G>A	GGGAA	0%	0%	0%	0%	0%	11%
2818	G>A	AAGAA	17%	29%	0%	0%	0%	0%
2821	G>A	AAGAC	0%	0%	0%	0%	0%	7%
2836	T>G	TTTAC	18%	31%	0%	0%	0%	0%
2850	T>A	CTTT	3%	17%	0%	0%	0%	0%
2851	T>C	CTTTG	9%	20%	0%	0%	0%	0%
2852	T>G	TTTGT	0%	5%	0%	0%	0%	0%

**Table 2:** RNA editing detection in the 28S rRNA gene from the NGS data and IGV view.

Editing Site in Transcript; Gene ID PF3D7_0112700	Editing Types	Genomic Sequence	Editing% in Time Course/NGS Data on IGV View			
			16 h	24 h	32 h	40 h
2737	T>G	ATTAA	20%	9%	3%	3%
2761	T>C	ACTTG	19%	7%	2%	3%
2763	G>A	TTGCT	20%	7%	2%	3%
2769	T>C	TCTAG	19%	8%	2%	4%

## Data Availability

The detailed data are available upon request to the corresponding author.

## Supplementary Materials

The following supporting information can be downloaded at: https://www.mdpi.com/article/10.3390/microorganisms12010137/s1.
