# Supplementary material for: Detection of Developmental Asexual Stage-Specific RNA Editing Events in Plasmodium falciparum 3D7 Malaria Parasite
Source: Microorganisms. 2024 Jan 10;12(1):137. doi: 10.3390/microorganisms12010137 (PMC10819399; doi:10.3390/microorganisms12010137)

Supplementary Figure S1. Azad *et al.*

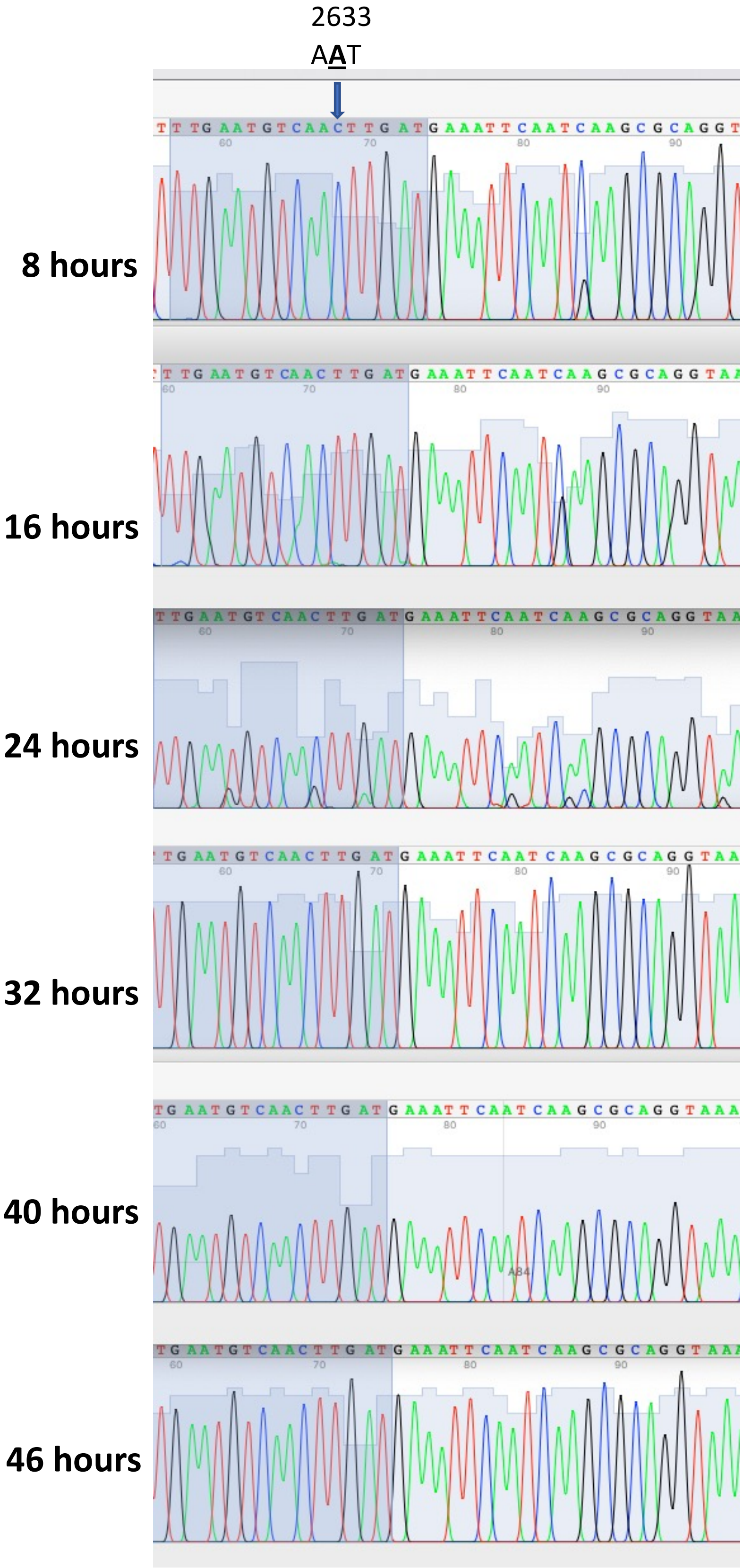

Chromosome 1 sequence TTGAATGTCAA**A**TTGAT. Genomic A>C, position 2633, 100% substitution due to copy number variation in 28S rRNA genes.
TTGAATGTCAA**C**TTGAT- mRNA.

Supplementary Figure S2. Azad *et al.*

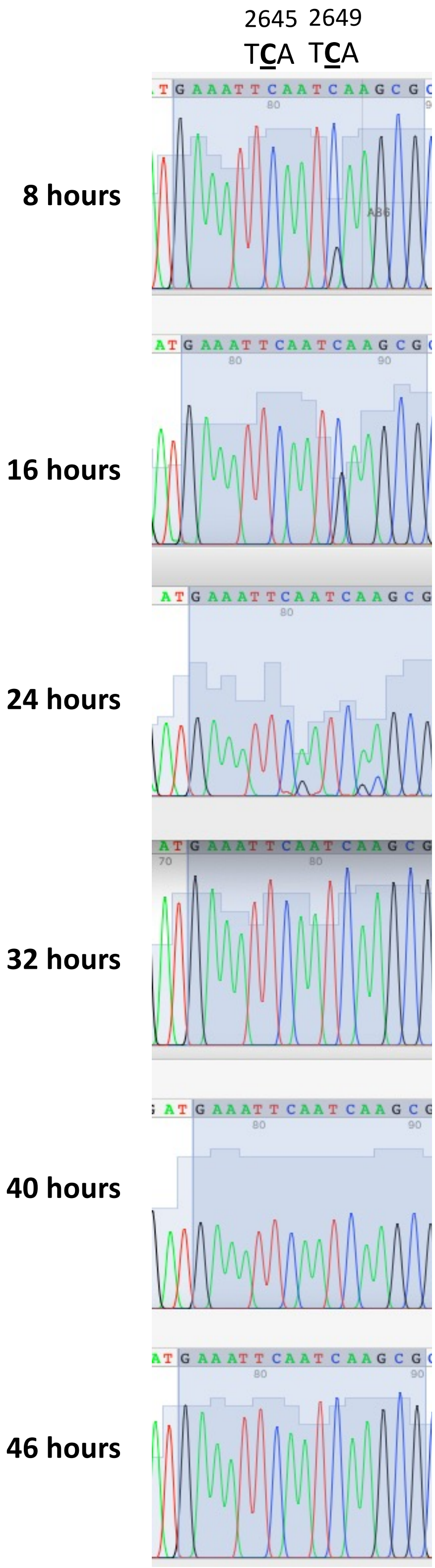

Chromosome 1 GAAATTTAATTAAGCG, Chromosome 5, and Chromosome 7 GAAATTCAATCAAGCGCA, T>C Complete substitution at 2645 and 2649 TTTAA>TTTAA, 2645 position C>T position 2649 complete substitution CAAGCGCA>AATGAAGC, 2649 position (C>G at position 2649, base conversion). Chromosome 5 and 7 appear much.

Supplementary Figure S3. Azad *et al.*

chromosome 5 GTTTCTATTCAGTACTTTC

chromosome 7 GTTTCTATACAGTACTTTC

GTTTCTATATAGTACTTTCT genomic sequence 729, 730 position in chromosome 1

Gradual changing of A>T and substitution of T by C

729 730

TTT TTA

8 hours

16 hours

24 hours

32 hours

40 hours

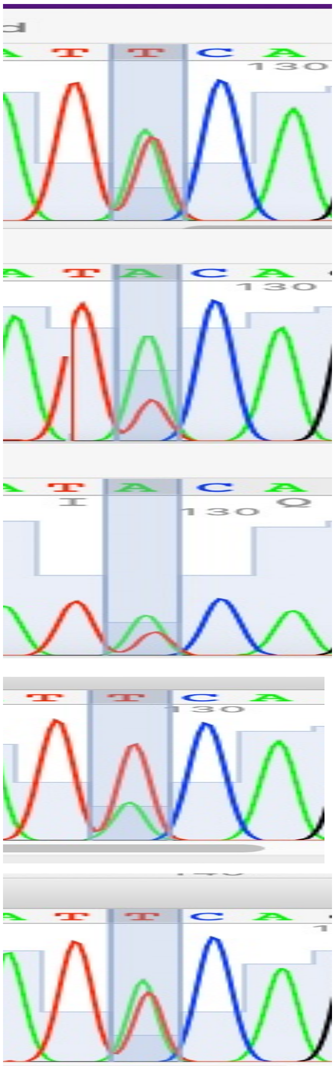

Chromosome 5, PF3D7_0532000 and chromisome 7, PF3D7_0726000. 28S rRNA are mainly expressed. PF3D7_0726000 dominant in the ring stage and PF3D7_0532000 in Trophozoite and Schizont stages. C is the genomic locus of only PF3D7_0726000 and PF3D7_0532000, indicating different 28S rRNA expression in the different developmental stages of *Plasmodium falciparum*. NGS expression data also show that these two 28S rRNA expression levels are higher than those of the other 28S rRNA genes. 46 hours, not detected.

Supplementary Figure S4. Azad *et al.*

PF3D7_1227200, potassium channel K1

TCAAAGAAAAAAAAAAAAAAAAAAAAAAAAGAGAA;
G>A predicted but absent in stage-specific results

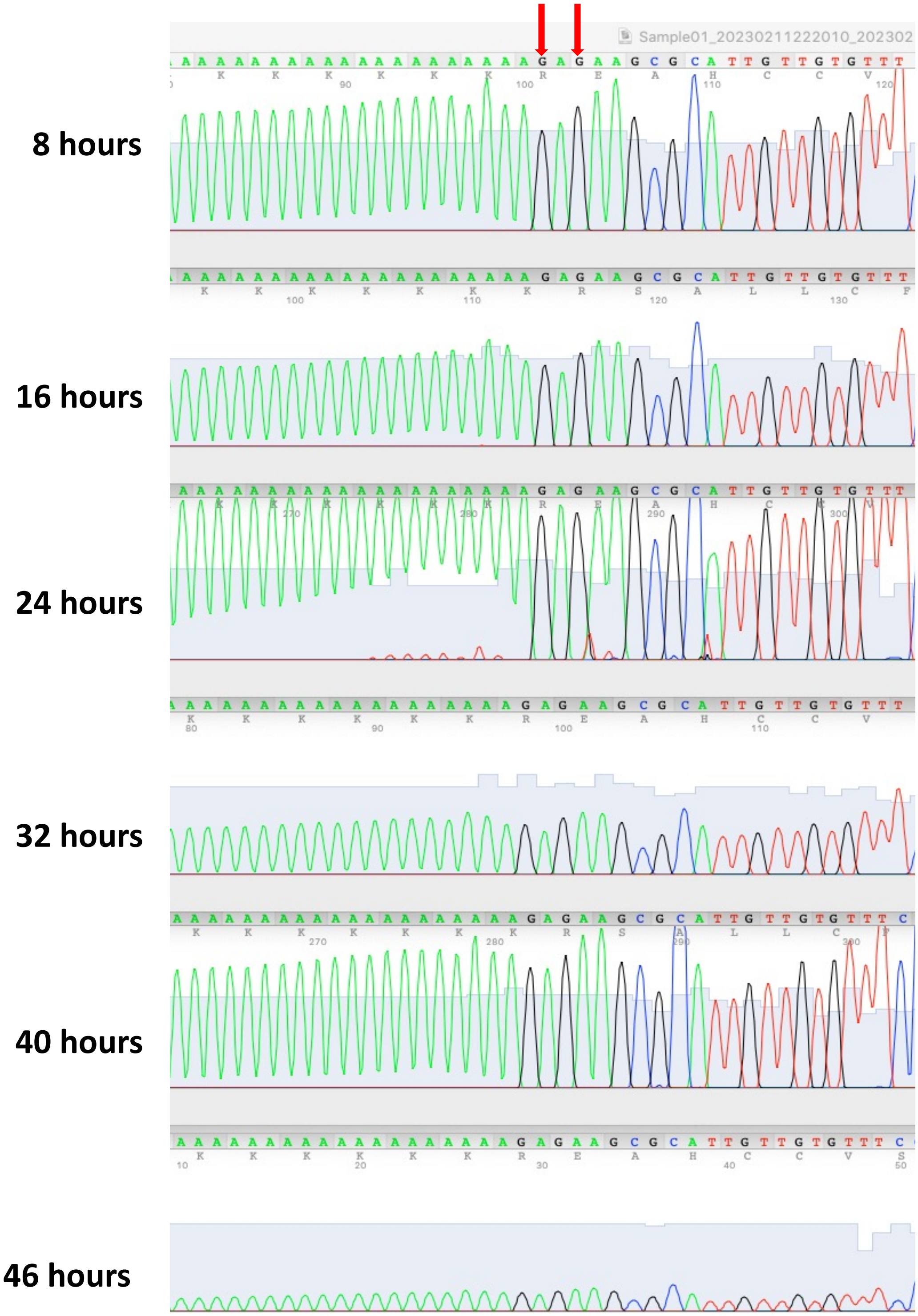

PF3D7_1149000;antigen 332, DBL-like protein (Not found)

ACTGTTAATGAA̲GAAATGCCCGTAG> ACTGTTAATGAA̲GAAATGCCCGTAG
4151st position A>G

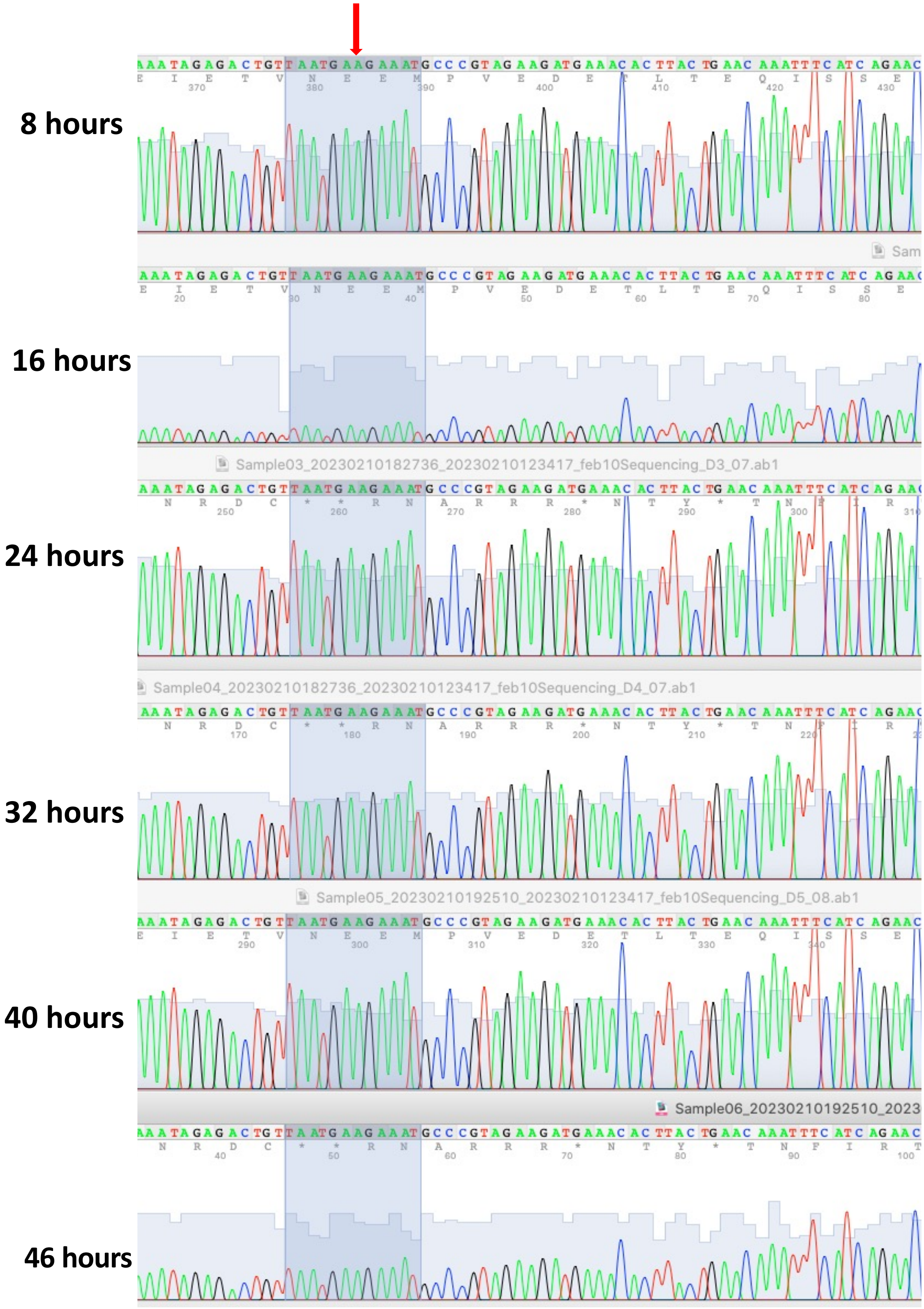

Supplementary Figure S6. Azad *et al.*

PF3D7_0815200/ importin subunit beta

ATTTTATACA> ATTTTAAACA, previously reported as SNV, (not found)

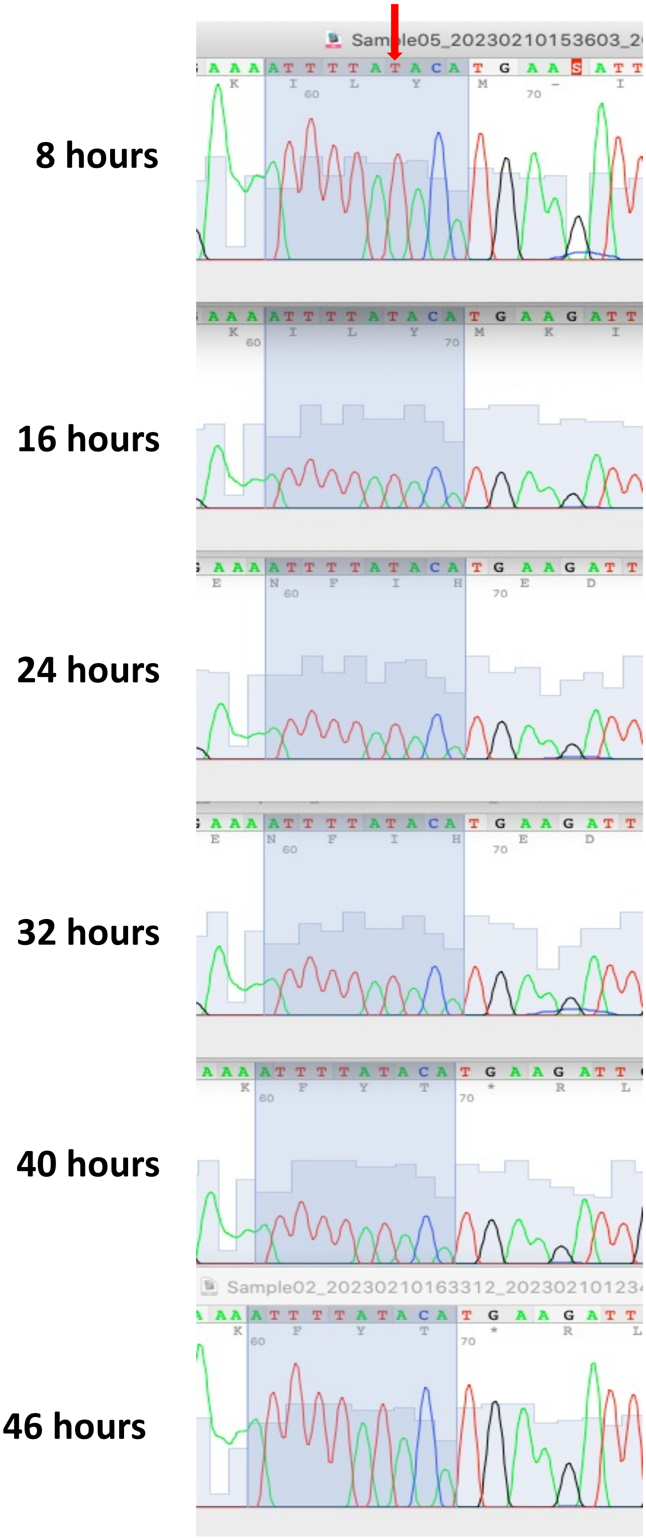

Supplementary Figure S7. Azad *et al.*

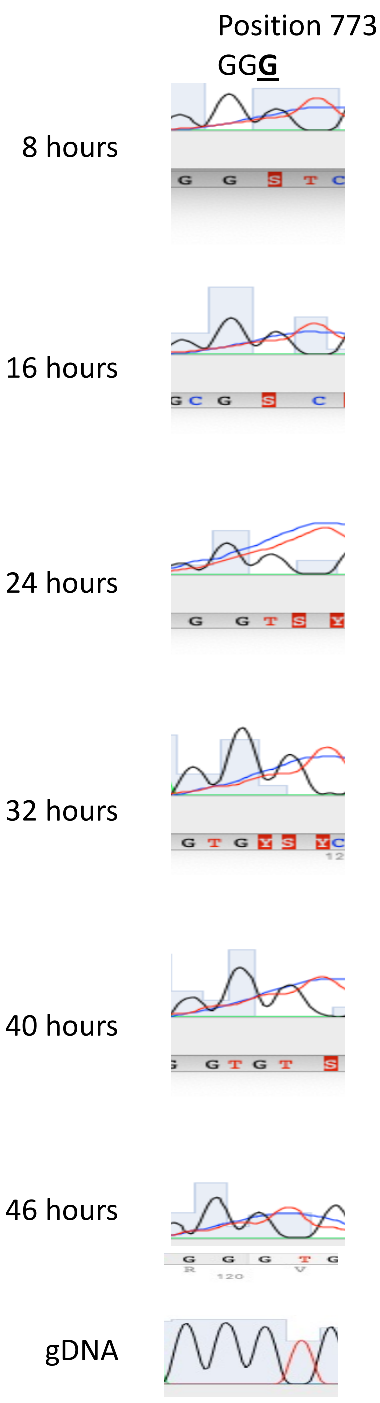

G>T/C editing in the all stages but not found in the gDNA.
Sanger Sequencing data of PF3D7_0216900

Supplementary Figure S8. Azad *et al.*

16 hours

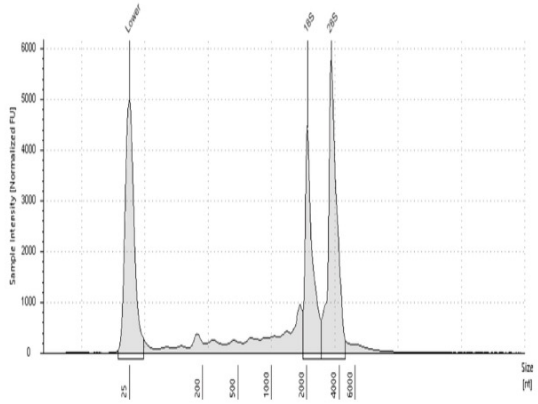

24 hours

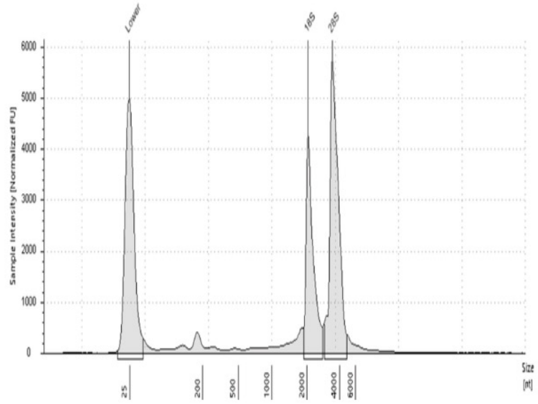

16 hours

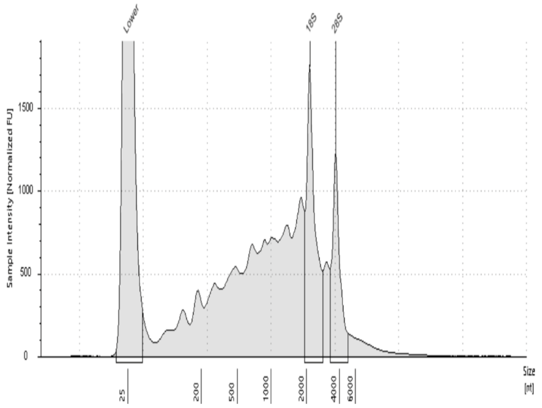

24 hours

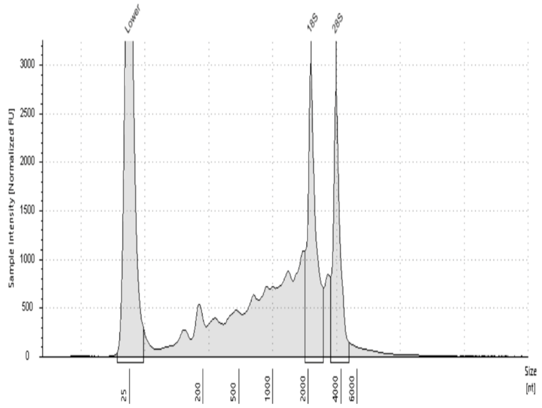

16 hours

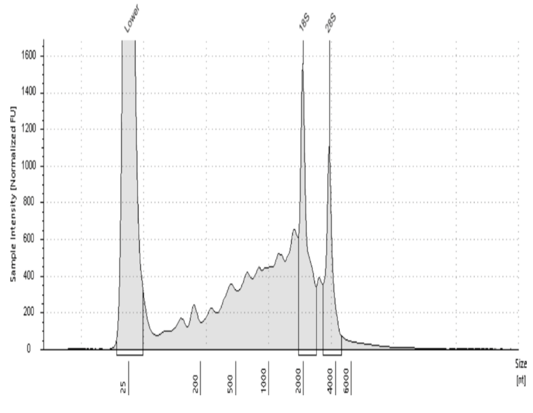

24 hours

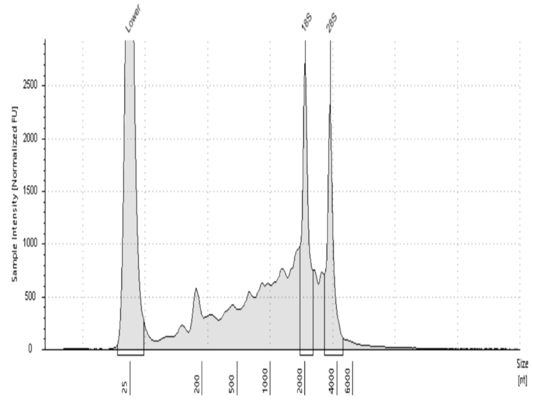

28S rRNA spectral peak present in all samples, indicating no hidden breaks and well integrated 28S rRNA.

Supplementary Figure S9. Azad *et al.*

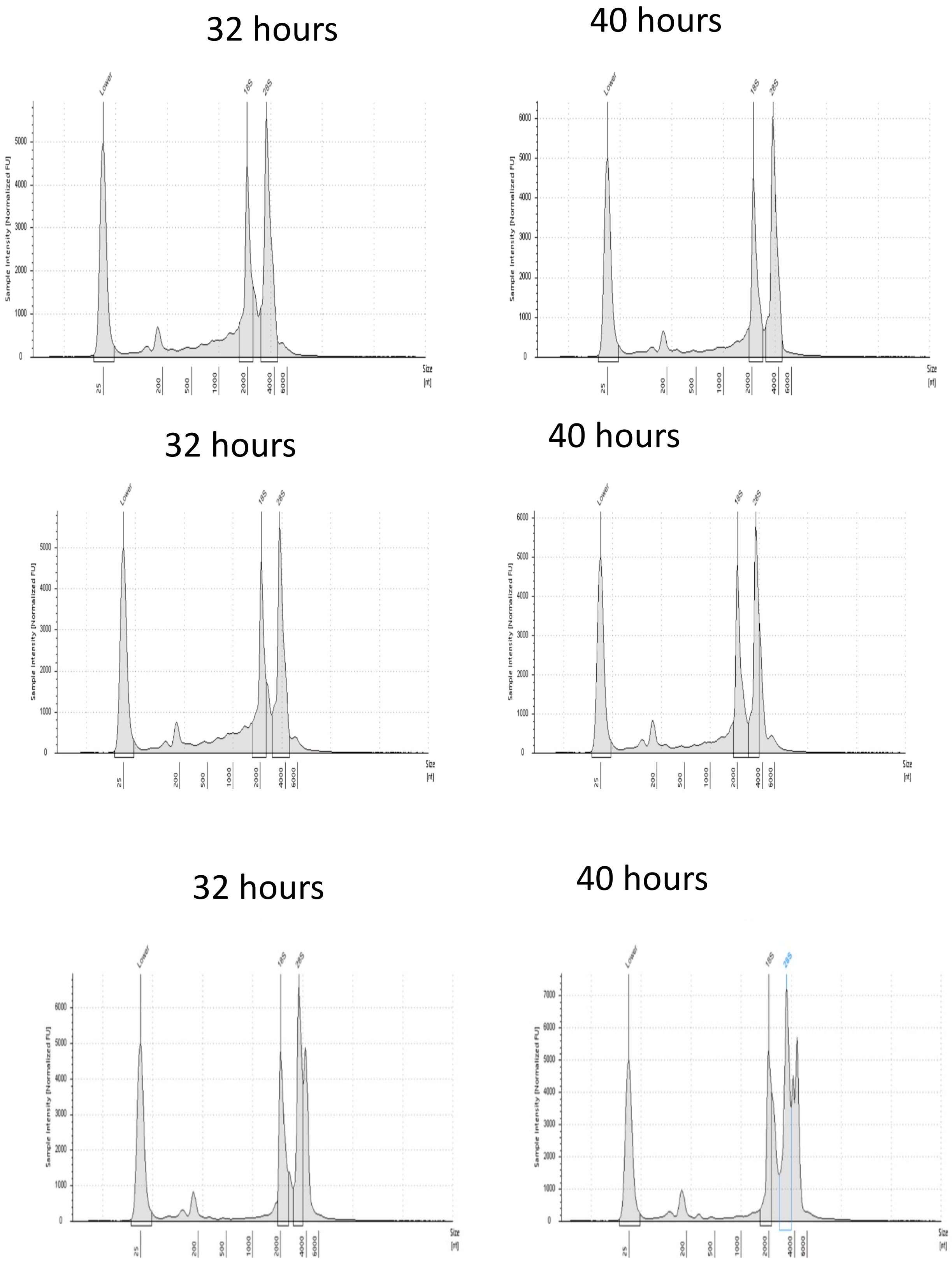

28S rRNA spectral peak present in all samples, indicating no hidden breaks and well integrated 28S rRNA.

Supplementary Figure S10. Azad *et al.*

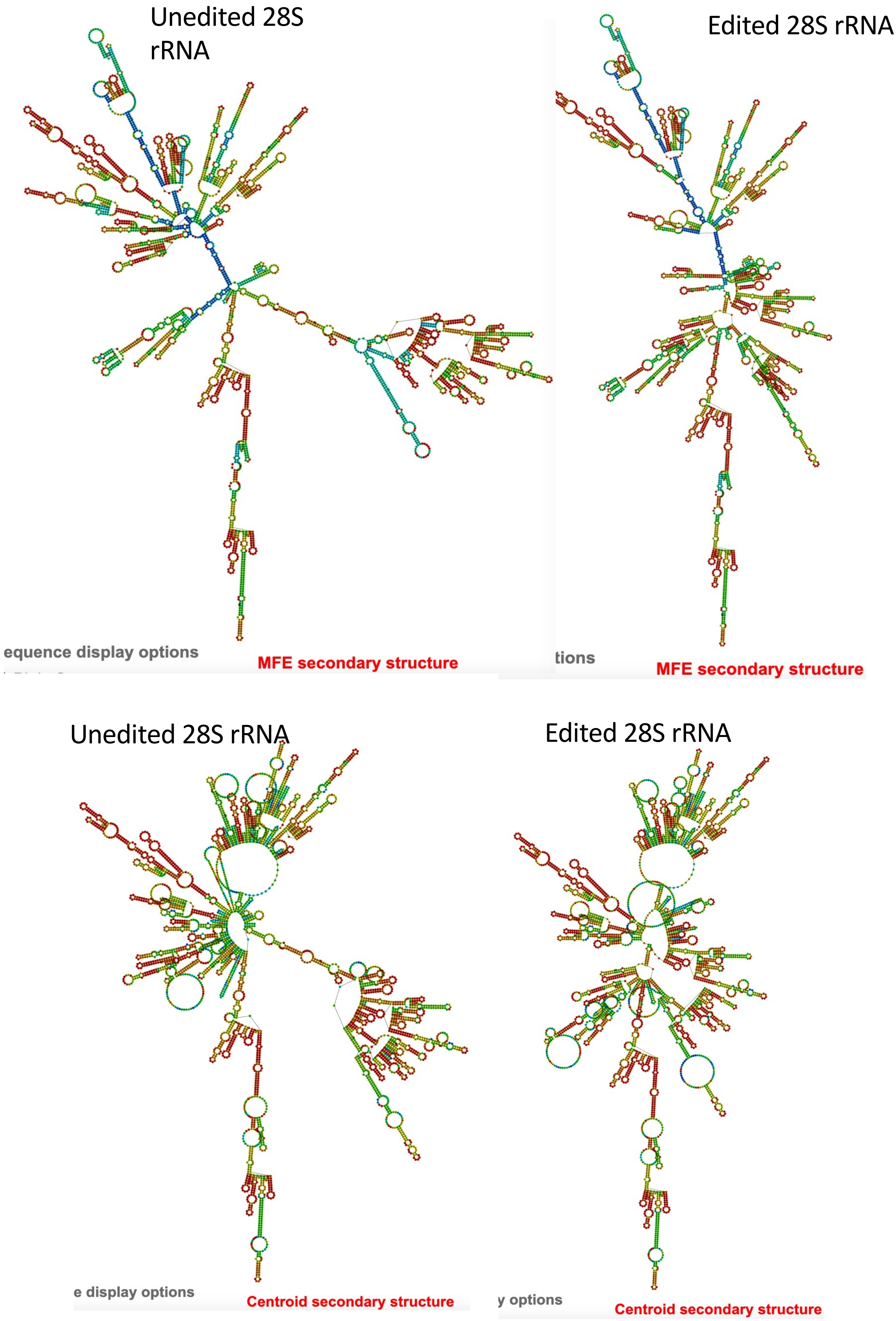

RNA secondary structure is altered due to RNA editing in 28S rRNA.

Supplementary Figure S11. Azad *et al.*

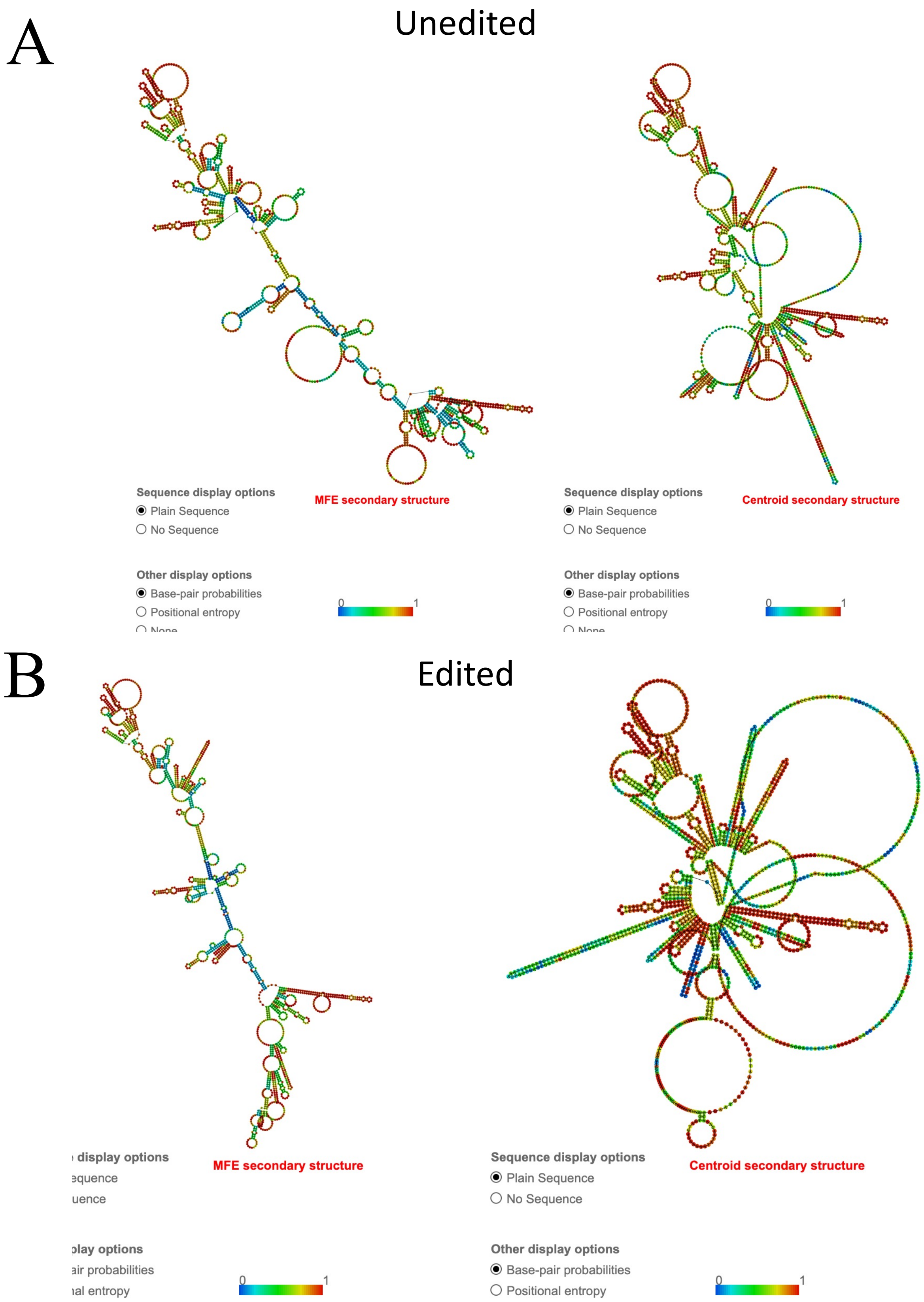

PF3D7_0216900 gene. (A) Unedited transcript forms a more complex structure than the edited one. (B) RNA editing transforms most of the loop structure to the stem structure.

Supplementary Figure S12. Azad *et al.*

A

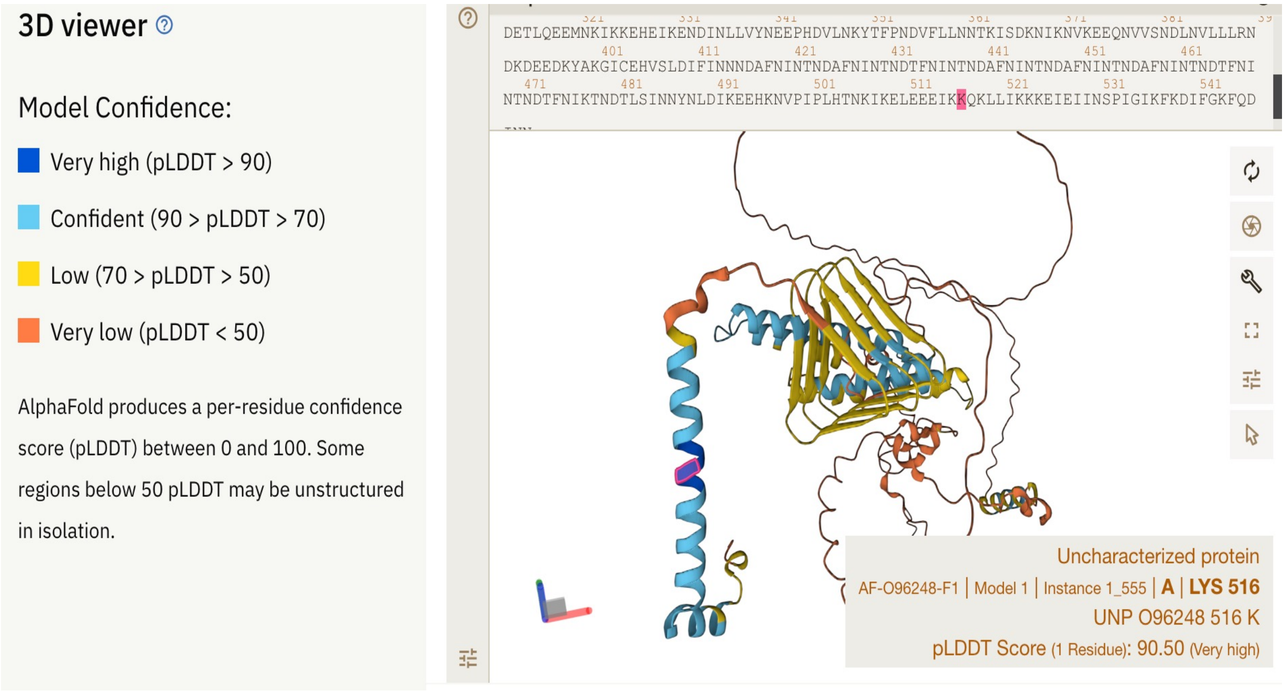

B

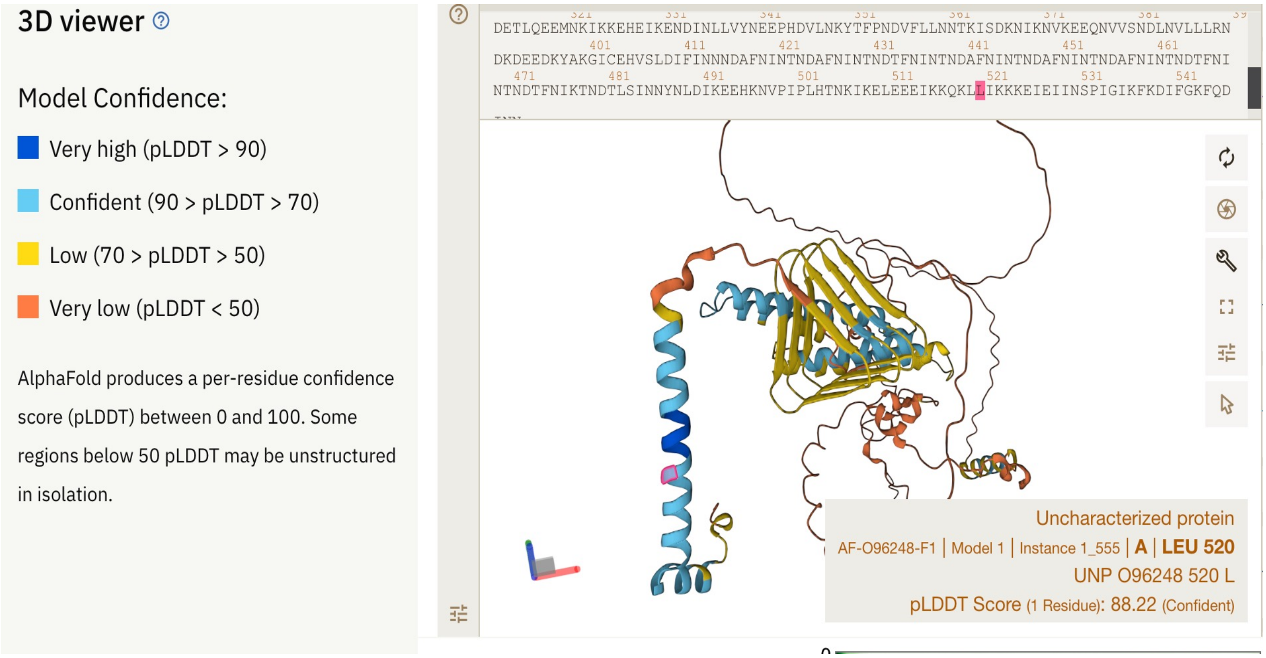

(A) Unedited protein residues 516 and 520 bind to hemoglobin. 516 pLDDT score is very high. (B) Unedited protein residues 516 and 520 bind to hemoglobin. 520 pLDDT score confident is high.

Supplementary Figure S13. Azad *et al*.

Consensus prediction of GO terms

**Molecular Function**	GO:0005515	GO:0008565								
**GO-Score**	0.45	0.35								
**Biological Process**	GO:0051246	GO:0009894	GO:0051707	GO:0033157	GO:0051168	GO:0050658	GO:0051298	GO:0046822	GO:0046605	GO:0033365
**GO-Score**	0.49	0.49	regulation of catabolic process	[illegible]	0.49	0.49	0.49	0.49	0.49	0.49
**Cellular Component**	GO:0016604	GO:0042175	GO:0032991	GO:0000775	GO:0005737					
**GO-Score**	0.49	0.49	0.49	0.49	0.35					

(a) Cscore[GO] is a combined measure for evaluating global and local similarity between query and template protein. It's range is [0-1] and higher values indicate more confident predictions.
(b) TM-score is a measure of global structural similarity between query and template protein.
(c) RMSD[a] is the RMSD between residues that are structurally aligned by TM-align.
(d) IDEN[a] is the percentage sequence identity in the structurally aligned region.
(e) Cov represents the coverage of global structural alignment and is equal to the number of structurally aligned residues divided by length of the query protein.

Unedited

Consensus prediction of GO terms

**Molecular Function**	GO:0008565	GO:0005515	GO:0003723							
**GO-Score**	0.62	0.48	0.48							
**Biological Process**	GO:0033365	GO:0009894	GO:0051707	GO:0051298	GO:0046822	GO:0046605	GO:0051246	GO:0033157	GO:0030163	GO:0006611
**GO-Score**	0.46	0.[illegible]	regulation of catabolic process	0.46	0.46	0.46	0.46	0.46	0.44	0.44
**Cellular Component**	GO:0005730	GO:0000776	GO:0005642	GO:0030529	GO:0015030	GO:0005829	GO:0005635			
**GO-Score**	0.48	0.40	0.40	0.40	0.40	0.33	0.33			

(a) Cscore[GO] is a combined measure for evaluating global and local similarity between query and template protein. It's range is [0-1] and higher values indicate more confident predictions.
(b) TM-score is a measure of global structural similarity between query and template protein.
(c) RMSD[a] is the RMSD between residues that are structurally aligned by TM-align.
(d) IDEN[a] is the percentage sequence identity in the structurally aligned region.
(e) Cov represents the coverage of global structural alignment and is equal to the number of structurally aligned residues divided by length of the query protein.
(f) The second table shows a consensus GO terms amongst the top scoring templates. The GO-Score associated with each prediction is defined as the average weight of the GO term, where the weights [illegible]

Edited

RNA editing in the PF3D7_0216900 gene of *Plasmodium falciparum*. GO term GO:0009894 responsible for catabolic process regulation.

Supplementary Figure S14. Azad *et al.*

16 h; 2737 T>G 13+30+18 average 20.33%

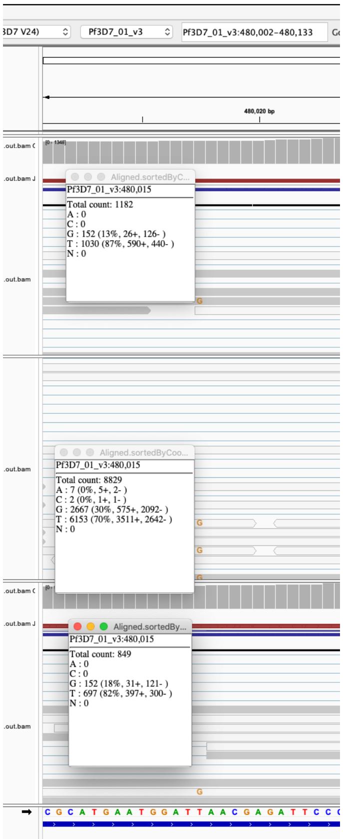

16 h; 2761 T>C 20+26+11 average 19%

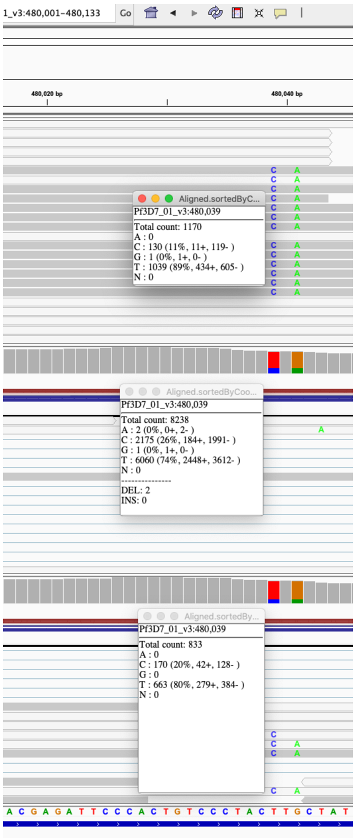

16 h; 2763 G>A 21+ 27+12 average 20%

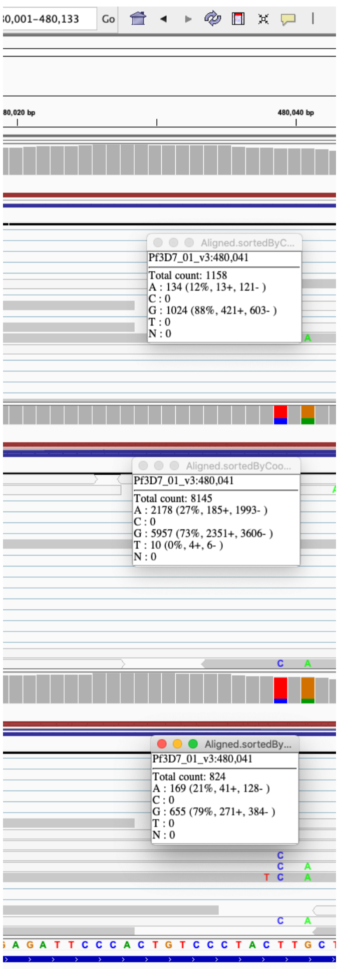

16 h; 2769 T to c 28+17+12 average 19%

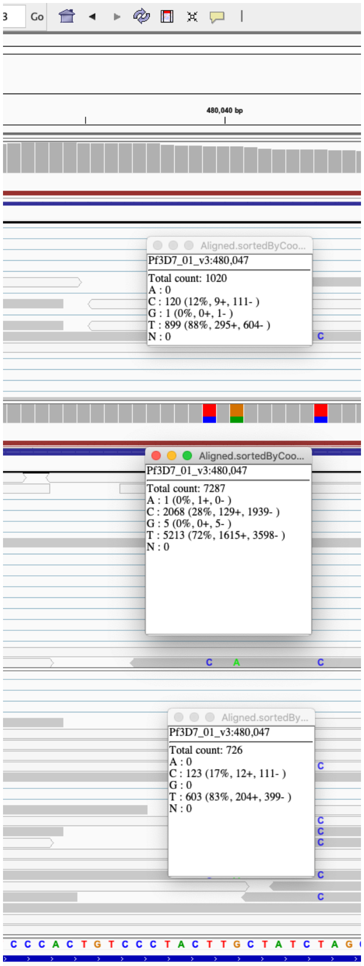

Supplementary Figure S15. Azad *et al.*

Position 2737;
24 hours T >G. 15+8+4=27/3=average 9%

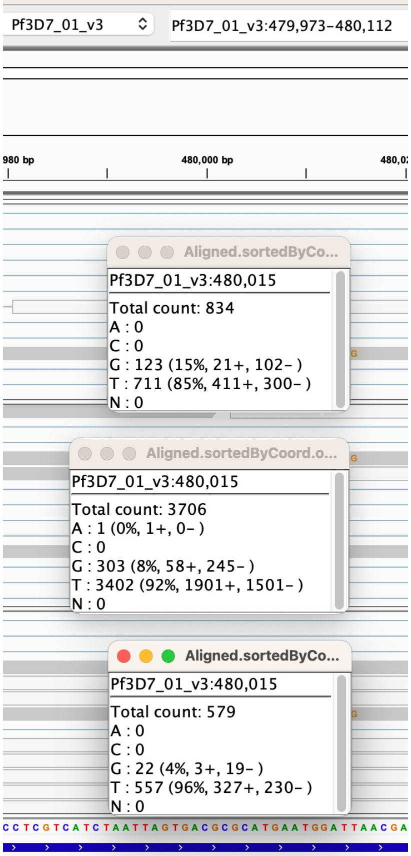

Position2761;
24 hours ; T>C 12+7+3=22/3= average7%

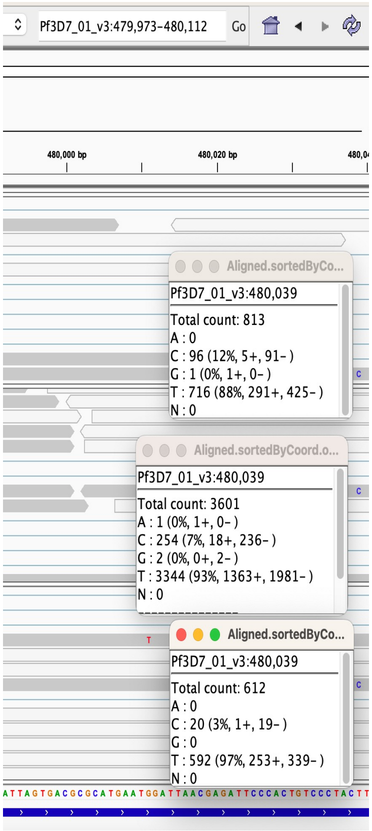

Position2763;
24 hours; G>A 12+7+3=22/3=average 7%

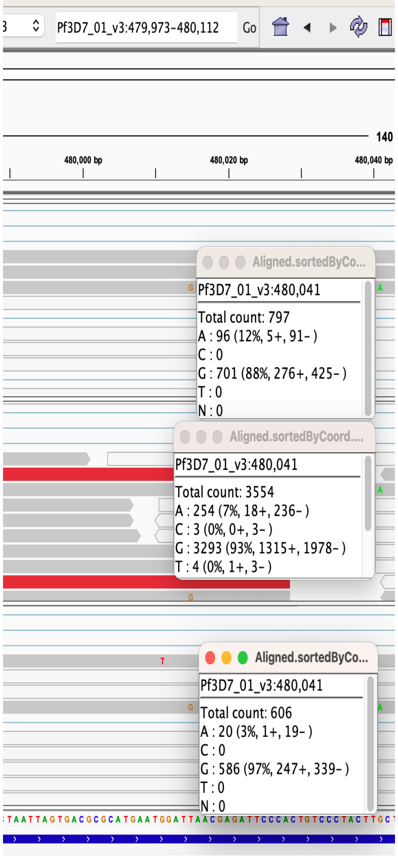

Position2769;
24 hours ; 2769 T>C 12+8+4=24/3=average 8%

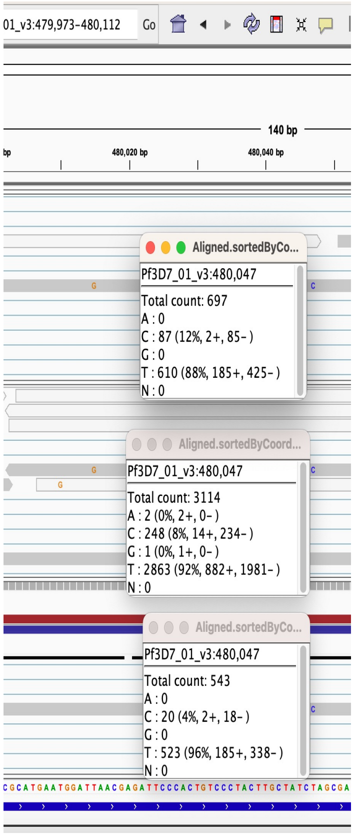

Supplementary Figure S16. Azad *et al.*

Position 2737; 32 Hours
T>G. 3+3+1=3% Average

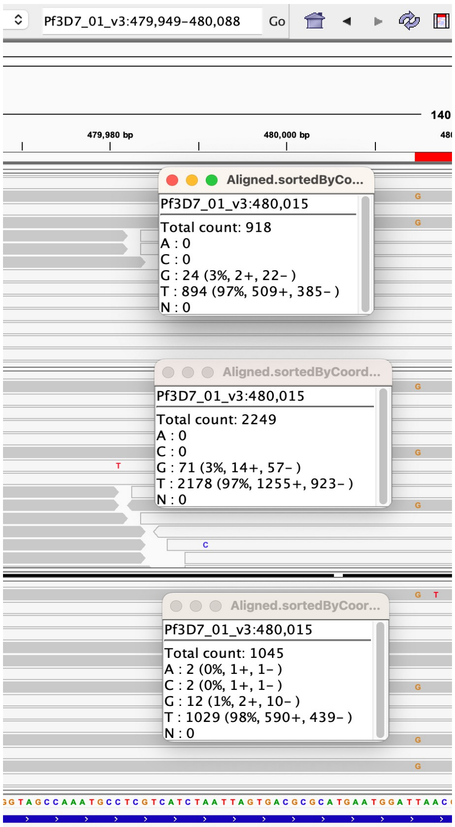

Position 2761; 32 Hours
T>C. 2+3+1=2% Average.

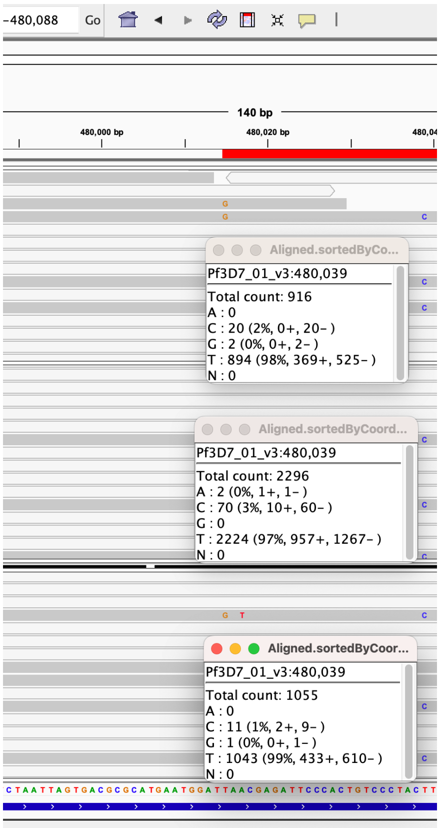

Position 2763; 32 Hours
G>A. 2+3+1=2% Average.

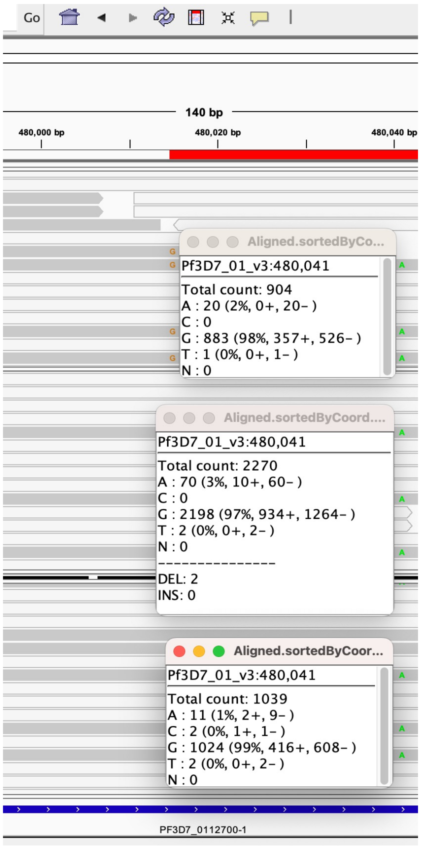

Position 2769; 32 Hours
T>C. 2+3+1=2% Average.

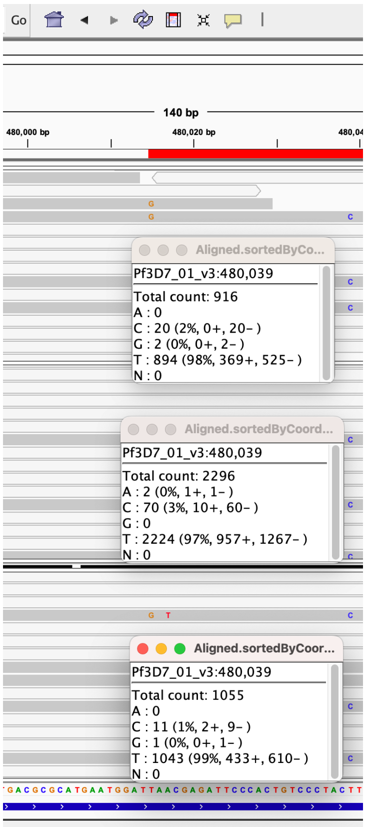

Supplementary Figure S17. Azad *et al.*

Position 2737, 40 hours T>G
8+1+1 average 3 %

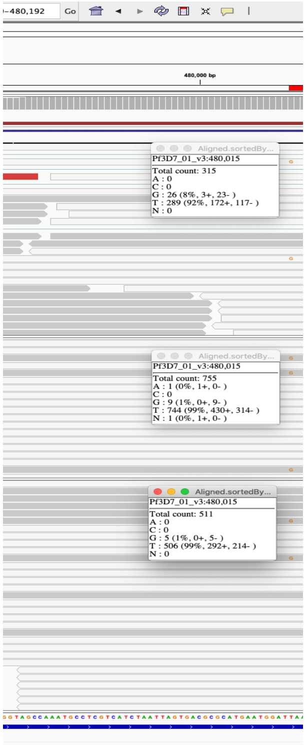

Positio 2761, 40 hours T>C 7+1+1;
average 3 %

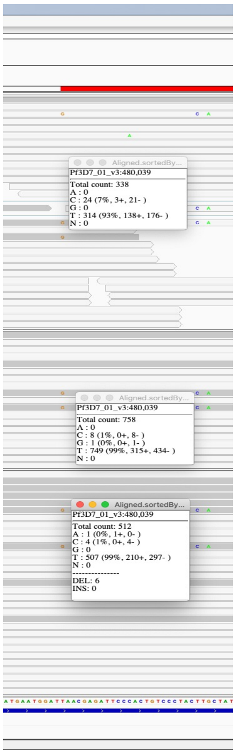

position, 2763
40hours G>A 7+1+1 ; average 3%

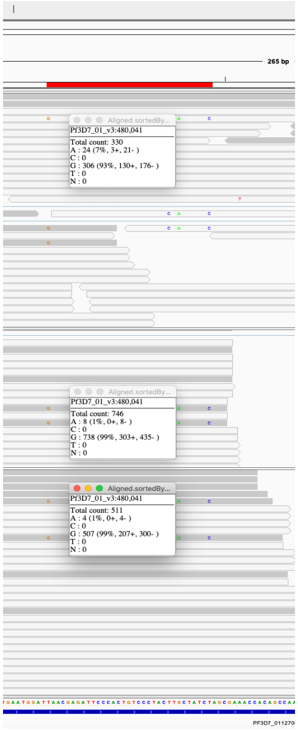

position,2769
40hours T>C 9+1+1; average 4%

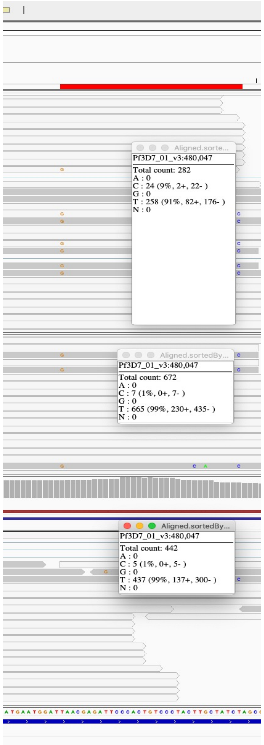

Supplement: Supplementary file 1 [file microorganisms-12-00137-s001.zip › Supplement Figures.pdf]
