# Supplementary material for: Detection of Developmental Asexual Stage-Specific RNA Editing Events in Plasmodium falciparum 3D7 Malaria Parasite
Source: Microorganisms. 2024 Jan 10;12(1):137. doi: 10.3390/microorganisms12010137 (PMC10819399; doi:10.3390/microorganisms12010137)
Supplement: Supplementary file 1 [file microorganisms-12-00137-s001.zip › Supplementary File S1.pdf]

CLUSTAL O(1.2.4) multiple sequence alignment of 28S rRNA genes

```
PF3D7_0532000,28S      -GTTTGATTTGATGTTAGGAGGGCAAATCCGCCGAATTTAAGCATATAATTAAGCGGAAG
        59
PF3D7_0726000,28S      -GTTTGATTTGATGTTAGGAGGGCAAATCCGCCGAATTTAAGCATATAATTAAGCGGAAG
        59
PF3D7_0112700,         ---------------TAGGAGGGCAAATCCGCTGAACTTAAGCATATAATTAAGCGGAAG
        45
PF3D7_1148640,         --TTTGATTTGATGTTAGGAAGGCAAATCCGCCGAATTTAAGCATATAATTAAGCGGAAG
        58
PF3D7_1371300,         TATTTGATTTGATGTTAGGAAGGCAAATCCGCCGAATTTAAGCATATAATTAAGCGGAAG
        60
                                      ***** *********** *** ***********************

PF3D7_0532000,28S      AAAAGAAAATAACTATGATTCCTTTAGTAACGGCGAGTGAACAAGGACCAGCTCAAACGG
        119
PF3D7_0726000,28S      AAAAGAAAATAACTATGATTCCTTTAGTAACGGCGAGTGAACAAGGACCAGCTCAAACGG
        119
PF3D7_0112700,         AAAAGAAAATAACTATGATTCCTTTAGTAACGGCGAGTGAAGAAGGAATAGCTCAATAAG
        105
PF3D7_1148640,         AAAAGAAAATAACAATGATTTCTTTAGTAACGGCGAGTGAACAAGAAAAAGCTCAAGGAG
        118
PF3D7_1371300,         AAAAGAAAATAACAATGATTTCTTTAGTAACGGCGAGTGAACAAGAAAAAGCTCAAGGAG
        120
                       ************* ****** ******************** *** *  *******   *

PF3D7_0532000,28S      TTAATCCTTCGTTTGGTGCAAACATTTCGACGG---------------------AATTGT
        158
PF3D7_0726000,28S      TTAATCCTTCGTTTGGTGCAAACCTTTCGACGG---------------------AATTGT
        158
PF3D7_0112700,         TAGAATCCTTCGAATCTTAATGATATATTTATACATAGAATAAAGAATTGAAGGAATTGT
        165
PF3D7_1148640,         ATAATTCTATATCGTAGTTGTAAAAAGACTATGTGTATAGAAATTGTTCTGTT--ATAAG
        176
PF3D7_1371300,         ATAATTCTATATCGTAGTTGTAAAAAGACTATGTGTATAGAAATTGTTCTGTT--ATAAG
        178
                          *  *                                                ** 

PF3D7_0532000,28S      C--GTTGAATT-----------ATTCCATGAATGCCTTTAATTTTGAGCAAAGTGAGCAG
        205
PF3D7_0726000,28S      C--GTTGAATT-----------ATTCCATGAATGCCTTTAAT-TTGAGCAAAGTGAGCAG
        204
PF3D7_0112700,         CAAGTTGAATTGTACTCTTGTAGGCCTCACAGGTGAAATGAATATATAGAAGTAAAGTAG
        225
PF3D7_1148640,         GAAGAAGTAATCTGAGAATATATGGGATTTATATATATATTTAGTTATCGAAATAAGTAG
        236
PF3D7_1371300,         GAAGAAGTAATCTGAGAATATATGGGATTTATATATATATTTAGTTATCGAAATAAGTAG
        238
                          *  * * *                   *             *     *    ** **

PF3D7_0532000,28S      GAATGCTTCCCCATAGAGGGTGAAAGGCCCATAGTTCTTTTTAA-------------TTT
        252
PF3D7_0726000,28S      GAATGCTTCCCCATAGAGGGTGAAAGGCCCATAGTTCTTTTTAA-------------TTT
        251
PF3D7_0112700,         GAATACTTCCTCATAGAGGGTGAAAGGCCCGTATCATATAT-------CATTTCGTGGGC
        278
PF3D7_1148640,         GAATACTTTCCCATAGAGGGTGAAAGGCCCGTAGTTAATAAATATATAAAGTTCTTTTTT
        296
PF3D7_1371300,         GAATACTTTCCCATAGAGGGTGAAAGGCCCGTAGTTAATAAATATATAAAGTTCTTTTTT
        298
                       **** *** * ******************* **     *
```

```
PF3D7_0532000,28S      ATTCATTGGAATTTTTCATGCCGAGTCGTGTTCTTTGAGATTGGAGCACTAATACGTGTG
        312
PF3D7_0726000,28S      ATTCATTGGAATTTTTCATGTCGAGTCGTGTTCTTTGAGATTGGAGCACTAATACGTGTG
        311
PF3D7_0112700,         TTTGGAGTATTTATATTTTACTGAGTAGTGTTCTTTGAGATTGGAGCACAAATTGGTGTG
        338
PF3D7_1148640,         TTTGGATAATTTTTTTTTTAAAGAGTAGTGCTCTTTGAGATTGGAGTACAAAGATGTGTG
        356
PF3D7_1371300,         TTTGGATAATTTTTTTTTTAAAGAGTAGTGCTCTTTGAGATTGGAGTACAAAGATGTGTG
        358
                        **        * * *  *   **** *** *************** ** **   *****

PF3D7_0532000,28S      ATACATTTCACATAAAGCTAAATATGTGTAGGAGACCGATAGCAAACAAGTACCGTGAGG
        372
PF3D7_0726000,28S      ATACATTTCACATAAAGCTAAATATATGTAGGAGACCGATAGCAAACAAGTACCGTGAGG
        371
PF3D7_0112700,         ATACATTTCACATAAAGCTAAATATGTACAGGAGACCGATAGCGAACAAGTACCGTGAGG
        398
PF3D7_1148640,         ATACATTTCACATAAGGCTAAATATATATAGGAGACCGATAGGAAACAAGTACCGTGAGG
        416
PF3D7_1371300,         ATACATTTCACATAAGGCTAAATATATATAGGAGACCGATAGGAAACAAGTACCGTGAGG
        418
                       *************** ********* *  *************  ****************

PF3D7_0532000,28S      GAAAGATGAAATAGTACTCAGGAATGAGCAATTAAATAGTACCTGAAATCGTTAAGATGG
        432
PF3D7_0726000,28S      GAAAGATGAAATAGTACTCAGGAATGAGCAATTAAATAGTACCTGAAATCGTTAAGATGG
        431
PF3D7_0112700,         GAAAGATGAAATAGTACTCAGGAATGAGCAATTAAATAGTACCTGAAATCGTTAAGATGG
        458
PF3D7_1148640,         GAAAGATGAAATAGTACTCAGGAATGAGCAGTTAAATAGTACCTGAAATCGTTAAGGTGG
        476
PF3D7_1371300,         GAAAGATGAAATAGTACTCAGGAATGAGCAGTTAAATAGTACCTGAAATCGTTAAGGTGG
        478
                       ****************************** ************************* ***

PF3D7_0532000,28S      AACGGATTAAGAGAGAAAACAAGTAAAGAGGGGAATTTTTAATTTTTTTTGTTA------
        486
PF3D7_0726000,28S      AACGGATTAAGAGAGAAAACAAGTAAAGAGGGGAATTTTTAATTTTTTTTGTTA------
        485
PF3D7_0112700,         AACGGATTAAGAGAGAAAACAAGTAAAGAGGAGAATTTTTTACATTTTTATTTTTGTTTT
        518
PF3D7_1148640,         AACGGATTAAGAAAGGAAAATGTAAGAAGATAGTAAATATTACTCGGCGAGTTTTATTAA
        536
PF3D7_1371300,         AACGGATTAAGAAAGGAAAATGTAAGAAGATAGTAAATATTACTCGGCGAGTTTTATTAA
        538
                       ************ ** ***     * *     * *  * * *         **

PF3D7_0532000,28S      -TAATTCTCTTCTTTATTAAAAGAAACATCAGTGATTAATTTAATTTCAA----------
        535
PF3D7_0726000,28S      -TAATTCTCTTCTTTATTAAAAGAAACATCAGTGATTAATTTAATTTCAA----------
        534
PF3D7_0112700,         ATAATTCTCTTCTTTATTAAAAGAAACATCAGTGATTAATTT-------------AATT-
        564
PF3D7_1148640,         GATACG-ATTTTTTTATAAAAAGAAACATGAGTGAACGAGAATAAATTATAATATAATTA
        595
PF3D7_1371300,         GATACG-ATTTTTTTATAAAAAGAAACATGAGTGAACGAGAATAAATTATAATATAATTA
        597
                          *     ** ***** *********** *****   *

PF3D7_0532000,28S      -----------------------------------------------------TAAAGCA
        542
```

```
PF3D7_0726000,28S      -----------------------------------------------------TAAAGCA
        541
PF3D7_0112700,         ------------------TCAA-------------------------------TAAAGCA
        575
PF3D7_1148640,         TATTAGATAGGTATAGAATAGATACACAGGACTAAGGGGGGAGGAGTAGTAAGAAAAAGG
        655
PF3D7_1371300,         TATTAGATAGGTATAGAATAGATACACAGGACTAAGGGGGGAGGAGTAGTAAGAAAAAGG
        657
                                                                             ***   

PF3D7_0532000,28S      ATCCCCTGA--------------------------AATTCAAAATTTCTTTTAATTTTGT
        576
PF3D7_0726000,28S      ATCCCCTGA--------------------------AATTCAAAATTTCTTTTAATTTTGT
        575
PF3D7_0112700,         ATCCCCTGA--------------------------AATTCAAAATTTCTTTTAATTTTGT
        609
PF3D7_1148640,         GTGCAGTGTAAAAGGGTTATGCTTGTATGTTTGAGTATTCACTGCTTCTTTTTTTTTTTT
        715
PF3D7_1371300,         GTGCAGTGTAAAAGGGTTATGCTTGTATGTTTGAGTATTCACTGCTTCTTTTTTTTTTTT
        717
                        * *  **                            *****    *******  **** *

PF3D7_0532000,28S      TTTCACTTTCTCCCCGC------------------------------ACTAATGTGGGGA
        606
PF3D7_0726000,28S      TTTCACTTTCTCCCCGC------------------------------ACTAATGTGGGGA
        605
PF3D7_0112700,         TTTCACTTTCTC-CCCG--------------------------CACTAA----TGTGGGGA
        639
PF3D7_1148640,         TCTGTAATTTTATATCTATTTAATTAAAGTATCCTTAGCTAATAATAATTTATGAAGGAA
        775
PF3D7_1371300,         TCTGTAATTTTATATCTATTTAATTAAAGTATCCTTAGCTAATAATAATTTATGAAGGAA
        777
                       * *    ** *                                    *    **  ** *

PF3D7_0532000,28S      AAACTGGCTTTATTT---------------------------------------------
        621
PF3D7_0726000,28S      AAACTGGCTTTATTT---------------------------------------------
        620
PF3D7_0112700,         AAACTGGCTTTATTT---------------------------------------------
        654
PF3D7_1148640,         GAACTTAATTTATATGAGTTTTAAAAGGCATGATTATATTGTATATTATATGATATATAT
        835
PF3D7_1371300,         GAACTTAATTTATATGAGTTTTAAAAGGCATGATTATATTGTATATTATATGATATATAT
        837
                        ****   ***** *                                             

PF3D7_0532000,28S      ------------------------CTTCAATTATTTTTTTTGCTGAGGAATTTT------
        651
PF3D7_0726000,28S      ------------------------CTTCAATTATTTTTTTTGCTGAGGAATTTT------
        650
PF3D7_0112700,         ------------------------CTTCAATTATTTTTTTTGCTGAGGAATTTT------
        684
PF3D7_1148640,         ATAATTATGTTTTAACCGAAATGCGTATAATTATTCTGTTTACTGAGGAAAGAGGTAAAC
        895
PF3D7_1371300,         ATAATTATGTTTTAACCGAAATGCGTATAATTATTCTGTTTACTGAGGAAAGAGGTAAAC
        897
                                                *  ******* * *** ********          

PF3D7_0532000,28S      ------------AAAATTATTTGAATTTTTCTTCTTATAATTTAAGTTGTTTCTATTCAG
        699
PF3D7_0726000,28S      ------------AAAATTATTTGAATTTTTCTTCTTATAATTTAAGTTGTTTCTATACAG
        698
```

```
PF3D7_0112700,          ------------AAAATTATTTGAATTTTTCTTCTTATAATTTAAGTTGTTTCTATATAG
      732
PF3D7_1148640,          AATAGTGTGAACTAAGAATGGGATATTTCTTTTTTCTGATTGTAAGTTGTTTTTAAATAG
      955
PF3D7_1371300,          AATAGTGTGAACTAAGAATGGGATATTTCTTTTTTCTGATTGTAAGTTGTTTTTAAATAG
      957
                                     **         **** * ** *   * * ********** **   **

PF3D7_0532000,28S       TACTTTCTTAACCCACTCGTCTTGAAACACGGACCAAGGAGTCTAGCAAATGTGCAAGTG
      759
PF3D7_0726000,28S       TACTTTCTTAACCCACTCGTCTTGAAACACGGACCAAGGAGTCTAGCAAATGTGCAAGTG
      758
PF3D7_0112700,          TACTTTCTTAACCCACTCGTCTTGAAACACGGACCAAGGAGTCTAGCAAATGTGCAAGTG
      792
PF3D7_1148640,          TACTTTTTTAACCCACTCGTCTTGAAACACGGACCAAGGAGTCTAACAAATGTGCGAGCG
      1015
PF3D7_1371300,          TACTTTTTTAACCCACTCGTCTTGAAACACGGACCAAGGAGTCTAACAAATGTGCGAGCG
      1017
                        ****** ************************************** ********* ** *

PF3D7_0532000,28S       TATATGATTCTTTAAACATTTCTCTT----TTTAATATACGCATAATTAATGTAATATGT
      815
PF3D7_0726000,28S       TATATGATTCTTTAAACATTTCTCTT----TTTAATATACGCATAATTAATGTAATATGT
      814
PF3D7_0112700,          TATATGATTCTTTAAACATTTCTCTT----TTTAATATACGCATAATTAATGTAATATGT
      848
PF3D7_1148640,          TATGTGATATGAATAAAAACATATATAAATTTTTAGTTACGCGTAATTAATGTGAATTTT
      1075
PF3D7_1371300,          TATGTGATATGAATAAAAACATATATAAATTTTTAGTTACGCGTAATTAATGTGAATTTT
      1077
                        *** ****      ** *       *    *** *  ***** ********** *  * *

PF3D7_0532000,28S       TTCTTTATTGTAGATTTGTGTGGTGTTTAATTTTTATTAAATCCCCACTTTGCATACAAT
      875
PF3D7_0726000,28S       TTTCTTTAT-TGTAGATTTGTGGTGTTTAATTTTTATTAAATCCCCACTTTGCATACAAT
      873
PF3D7_0112700,          TTCTTTATTGTAGA--TTTGTGGTGTTTAATTTTTATTAAATCCCCACTTTGCATACAAT
      906
PF3D7_1148640,          TTGTGGATG---------CGCCGGA-CAATAGTGCATTTGGTTTCTGGCTTGCAAACAAT
      1125
PF3D7_1371300,          TTGTGGATG---------CGCCGGA-CAATAGTGCATTTGGTTTCTGGCTTGCAAACAAT
      1127
                        **                 *  *     *   *  ***   *  *    ***** *****

PF3D7_0532000,28S       --------ACCGGTAAGCAATTATGCTTTATTGAGTACGAGCATATTTGGTAGGACCCGA
      927
PF3D7_0726000,28S       --------ACCGGTAAGCAATTATGCTTTATTGAGTACGAGCATATTTGGTAGGACCCGA
      925
PF3D7_0112700,          --------ACCGGTAAGCAATTATGCTTTATTGAGTACGAGCATATTTGGTAGGACCCGA
      958
PF3D7_1148640,          ACCGATAAGCAATAAGATAATTAAGTTTATTTGAGTATGAGTACATTTGTTAGGACCCGA
      1185
PF3D7_1371300,          ACCGATAAGCAATAAGATAATTAAGTTTATTTGAGTATGAGTACATTTGTTAGGACCCGA
      1187
                                 *    *   ***** * **  ******* *** * ***** **********

PF3D7_0532000,28S       GAGGCTTTGAACTAAGCGTGATGAGATTGAAGTCAGACGAAAGTCTGATGGAGGATCGAG
      987
PF3D7_0726000,28S       GAGGCTTTGAACTAAGCGTGATGAGATTGAAGTCAGACGAAAGTCTGATGGAGGATCGAG
      985
PF3D7_0112700,          GAGGCTTTGAACTAAGCGTGATGAGATTGAAGTCAGGCGAAAGTCTGATGGAGGATCGAG
      1018
```

```
PF3D7_1148640,          GAGGCTTTGAACTAAGCGTGATGAGATTGAAGTCAGACGAAAGTCTGATGGAGGATCGAG
       1245
PF3D7_1371300,          GAGGCTTTGAACTAAGCGTGATGAGATTGAAGTCAGACGAAAGTCTGATGGAGGATCGAG
       1247
                        ************************************ ***********************

PF3D7_0532000,28S       TTGATACTGACGTGCAAATCGTTCATTTCAATCACGTTTAGGGGCGAAAGACTAATCGAA
       1047
PF3D7_0726000,28S       TTGATACTGACGTGCAAATCGTTCATTTCAATCACGTTTAGGGGCGAAAGACTAATCGAA
       1045
PF3D7_0112700,          TTGATACTGACGTGCAAATCGTTCATTTCAATCACGTTTAGGGGCGAAAGACTAATCGAA
       1078
PF3D7_1148640,          TTGATACTGACGTGCAAATCGTTCATTACAATCACGTTTAGGGGCGAAAGACTAATCGAA
       1305
PF3D7_1371300,          TTGATACTGACGTGCAAATCGTTCATTACAATCACGTTTAGGGGCGAAAGACTAATCGAA
       1307
                        *************************** ********************************

PF3D7_0532000,28S       AAGCCTATTAGCTGGTTATTTTCGAAAGATCTCTCAGGATCGCTGGAGTTGATTA--ATA
       1105
PF3D7_0726000,28S       AAGCCTATTAGCTGGTTATTTTCGAAAGATCTCTCAGGATCGCTGGAGTTGATTA--ATA
       1103
PF3D7_0112700,          AAGCCTATTAGCTGGTTATTTTCGAAAGATCTCTCAGGATCGCTGGAGTTGATTA--ATA
       1136
PF3D7_1148640,          AAGCCTATTAGCTGGTTATTTTCGAAAGATCTCTCAGGATCGCTGGAGTTGAGTTGATTA
       1365
PF3D7_1371300,          AAGCCTATTAGCTGGTTATTTTCGAAAGATCTCTCAGGATCGCTGGAGTTGAGTTGATTA
       1367
                        **************************************************** *    **

PF3D7_0532000,28S       TAATTTTACCAGGTAGAGATAATGATTAGAGGAATCAGGGAAAT-CGTTTTCCTTGACCT
       1164
PF3D7_0726000,28S       TAATTTTACCAGGTAGAGATAATGATTAGAGGAATCAGGGAAAT-CGTTTTCCTTGACCT
       1162
PF3D7_0112700,          TAATTTTACCAGGTAGAGATAATGATTAGAGGATTTAGGGAAATGAATTTTCCTTAACCT
       1196
PF3D7_1148640,          TAATTTTATAAGGTAGAGACAATGATTAGAGGGTTTAGGGGATTAAATATTTCTTAACCT
       1425
PF3D7_1371300,          TAATTTTATAAGGTAGAGACAATGATTAGAGGGTTTAGGGGATTAAATATTTCTTAACCT
       1427
                        ********  ********* ************  * **** * *   * ** *** ****

PF3D7_0532000,28S       ATTCTCAAACTCCCAATAGGTAAAAAGGGCGAAATAACTCAACTTTGTATT---TCACTC
       1221
PF3D7_0726000,28S       ATTCTCAAACTCCCAATAGGTAAAAAGGGCGAAATAACTCAA-TTTGTATT---TCACTC
       1218
PF3D7_0112700,          ATTCTCAAACTTCCAATATGTAAAAAGGGTGATATATCTGAATTGTGTGTATTTCACTCT
       1256
PF3D7_1148640,          ATTCTCAAACTTCCAATATGTAAAGAGGGTGCGATATCTGAAAAG----TATTTCACTTT
       1481
PF3D7_1371300,          ATTCTCAAACTTCCAATATGTAAAGAGGGTGCGATATCTGAAAAG----TATTTCACTTT
       1483
                        *********** ****** ***** **** *  *** ** **       *

PF3D7_0532000,28S       ATATTAAATAAAATAACTCCAAGTGGGCCATTTTTGGTAAGCAGAACTGGCGATGAGGGA
       1281
PF3D7_0726000,28S       ATATTAAATAAAATAACTCCAAGTGGGCCATTTTTGGTAAGCAGAACTGGCGATGAGGGA
       1278
PF3D7_0112700,          GTTTTAAATAAAATAACTCCAAGTGGGCCATTTTTGGTAAGCAGAACTGGCGATGAGGGA
       1316
PF3D7_1148640,          GGCGTAAATAAGATAACTCCAAGTGGGCCATTTTTGGTAAGCAGAACTGGCGATGAGGGA
       1541
```

```
PF3D7_1371300,          GGCGTAAATAAGATAACTCCAAGTGGGCCATTTTTGGTAAGCAGAACTGGCGATGAGGGA
        1543
                            ******* ************************************************

PF3D7_0532000,28S       TGCTCCTAACGCTTGGATAAGGTGCCTAA-ATATTCGCTCATGAGATCCCATAAAAGGTG
        1340
PF3D7_0726000,28S       TGCTCCTAACGCTTGGATAAGGTGCCTAA-ATATTCGCTCATGAGATCCCATAAAAGGTG
        1337
PF3D7_0112700,          TGCTCCTAACGCTTGGATAAGGTGCCTAAATTATTCGCTCATGAGATCCCATAAAAGGTG
        1376
PF3D7_1148640,          TGCTCCTAACGCCTGGATAAGGTGCCTAA-ATATTCGCTCATGAGATCCCATAAAAGGTG
        1600
PF3D7_1371300,          TGCTCCTAACGCCTGGATAAGGTGCCTAA-ATATTCGCTCATGAGATCCCATAAAAGGTG
        1602
                        ************ ****************  *****************************

PF3D7_0532000,28S       TTGGTTCATAATGACAGTAGGACGGTGGTCATGGAAGTCGAAATCCGCTAAGGAGTGTGT
        1400
PF3D7_0726000,28S       TTGGTTCATAATGACAGTAGGACGGTGGTCATGGAAGTCGAAATCCGCTAAGGAGTGTGT
        1397
PF3D7_0112700,          TTGGTTCATAATGACAGTAGGACGATGGTCATGGAAGTCGAAATTCGCTTAGGAGTGTGT
        1436
PF3D7_1148640,          TTGGTTCATAATGACAGTAGGACGATGGTCATGGAAGTCGAAATTCGCTTAGGAGTGTGT
        1660
PF3D7_1371300,          TTGGTTCATAATGACAGTAGGACGATGGTCATGGAAGTCGAAATTCGCTTAGGAGTGTGT
        1662
                        ************************ ******************* **** **********

PF3D7_0532000,28S       AACAACTCACCTACCGAATGAACTAGCCCTGAAAATGGATGGCGCTAAAGCGGATTACCG
        1460
PF3D7_0726000,28S       AACAACTCACCTACCGAATGAACTAGCCCTGAAAATGGATGGCGCTAAAGCGGATTACCG
        1457
PF3D7_0112700,          AACAACTCACCTACCGAATGAACTAGCCCTGAAAATGGATGGCGCTAAAGCGAATTACCG
        1496
PF3D7_1148640,          AACAACTCACCTACCGAATGAACTAGCCCTGAAAATGGATGGCGCTAAAGCGAATTACCG
        1720
PF3D7_1371300,          AACAACTCACCTACCGAATGAACTAGCCCTGAAAATGGATGGCGCTAAAGCGAATTACCG
        1722
                        **************************************************** *******

PF3D7_0532000,28S       ATACCAAGCCATAAAAAGAACAGAAAAA--ATTTATTTTTTTTTAGAATCTTTTTATGA
        1518
PF3D7_0726000,28S       ATACCAAGCCATAAAAAGAACAGAAAAA--ATTTATTTTTTTTTAGAATCTTTTTATGA
        1515
PF3D7_0112700,          ATACCAGGCCATAAAAAGGTAGGAATTAAAATTTTAATTTACCTCAGATCTTTTTATGA
        1556
PF3D7_1148640,          ATACCGGGCCATAAGAAGGTAGAAATTATAAATGTTAATTTAGCTCAGATCTTTTTATGA
        1780
PF3D7_1371300,          ATACCGGGCCATAAGAAGGTAGAAATTATAAATGTTAATTTAGCTCAGATCTTTTTATGA
        1782
                        *****  ******* ***     **  *  * *  *  ***   *   ************

PF3D7_0532000,28S       GTAGAAAATCGTGGGGTTTGTGTTGAAGCGAAATACGTGAGTTTTCGTGGAACATCTCCC
        1578
PF3D7_0726000,28S       GTAGAAAATCGTGGGGTTTGTGTTGAAGCGAAATACGTGAGTTTTCGTGGAACATCTCCC
        1575
PF3D7_0112700,          GTAGAAAATCGTGGGGTTTGTGTTGAAGCGAAATACGTGAGTTTTCGTGGAACATCTCCC
        1616
PF3D7_1148640,          GTAGAAAATCGTGGGGTTTGTGTTGAAGCGAAATACGTGAGTTTTCGTGGAACATCTCCC
        1840
PF3D7_1371300,          GTAGAAAATCGTGGGGTTTGTGTTGAAGCGAAATACGTGAGTTTTCGTGGAACATCTCCC
        1842
                        ************************************************************
```

PF3D7_0532000,28S TAGTGCAGATCTTGGTGGAAGTAGCAACTATTCAAATGAGAACTTTGAAGACTGAAGTGG
1638
PF3D7_0726000,28S TAGTGCAGATCTTGGTGGAAGTAGCAACTATTCAAATGAGAACTTTGAAGACTGAAGTGG
1635
PF3D7_0112700, TAGTGCAGATCTTGGTGGAAGTAGCAACTATTCAAATGAGAACTTTGAAGACTGAAGTGG
1676
PF3D7_1148640, TAGTGCAGATCTTGGTGGAAGTAGCAACTATTCAAATGAGAACTTTGAAGACTGAAGTGG
1900
PF3D7_1371300, TAGTGCAGATCTTGGTGGAAGTAGCAACTATTCAAATGAGAACTTTGAAGACTGAAGTGG
1902
****

PF3D7_0532000,28S AGAAGGGTTTCTTGTCAACTGTGATTGAACAAGAGTTAGCCGCTCCTAAGGGATAGCTGA
1698
PF3D7_0726000,28S AGAAGGGTTTCTTGTCAACTGTGATTGAACAAGAGTTAGCCGCTCCTAAGGGATAGCTGA
1695
PF3D7_0112700, AGAAGGGTTTCTTGTCAACTGTGATTGAACAAGAGTTAGCCGCTCCTAAGGGATAGCTGA
1736
PF3D7_1148640, AGAAGGGTTTCTTGTCAACTATGTTTGTACAAGAGTTAGCCACTCCTAAGGGATAGCTGA
1960
PF3D7_1371300, AGAAGGGTTTCTTGTCAACTATGTTTGTACAAGAGTTAGCCACTCCTAAGGGATAGCTGA
1962
******************** ** *** ************* ******************

PF3D7_0532000,28S AAAGTGTTTAAAAG-GGGTTCCT------------------------TCCCCGTCTCAAA
1733
PF3D7_0726000,28S AAAGTGTTTAAAAGGGGGTTCCT------------------------TCCCCGTCTCAAA
1731
PF3D7_0112700, AAAGTGTTTAAAAGAAGAAATTCATTATAAGAAT--TATATAATGAAACTTCATCTCGAA
1794
PF3D7_1148640, AAAGTGTTTAAAAGAAGTAAGATATTATATGGAAATATATAATTAGATCTTCGTCTCAAA
2020
PF3D7_1371300, AAAGTGTTTAAAAGAAGTAAGATATTATATGGAAATATATAATTAGATCTTCGTCTCAAA
2022
************** *   * * **** **

PF3D7_0532000,28S AGGGAAACAGGTTGATATTCCTGTGCCAATAGTATTA--------TGAGTTTCTTAGATG
1785
PF3D7_0726000,28S AGGGAAACAGGTTGATATTCCTGTGCCAATAGTATTA--------TGAGTTTCTTAGATG
1783
PF3D7_0112700, AGGGAAACAGGTTAATATTCCTGTGCCATAAGTAATA-----AGAGTGCAAACAGAGATG
1849
PF3D7_1148640, AGGGAAACAGGTTAATATTCCTGTGCCAAATAGAAGTAAGGGTGTGAATAAAGAAAGATG
2080
PF3D7_1371300, AGGGAAACAGGTTAATATTCCTGTGCCAAATAGAAGTAAGGGTGTGAATAAAGAAAGATG
2082
************* **************   *   *****

PF3D7_0532000,28S GTAACATATATATAAATGAACTCCTTTACATAGGCTTTACACTCGGGGTGCGTTTTCTTT
1845
PF3D7_0726000,28S GTAACATATATATAAATGAACTCCTTTACATAGGCTTTACACTCGGGGTGCGTTTTCTTT
1843
PF3D7_0112700, GTAACATACATATAAATGAACTCCTTAACATAGATTTTACTCATGGGGTGCGTTATCTTT
1909
PF3D7_1148640, GTAACATACATACAAATGAACTCCTTGACATAGGTTTTACGCTCGGGGTGCGTTATCTTT
2140
PF3D7_1371300, GTAACATACATACAAATGAACTCCTTGACATAGGTTTTACGCTCGGGGTGCGTTATCTTT
2142
******** *** ************* ****** ***** * ********** *****

PF3D7_0532000,28S GCACTTTACCTTTATAACAAACCTTGGAATCAATTTACTTGGAGAAGAGGTTCGTTGAAC
1905

```
PF3D7_0726000,28S      GCACTTTACCTTTATAACAAACCTTGGAATCAATTTACTTGGAGAAGAGGTTCGTTGAAC
        1903
PF3D7_0112700,         GCACTTTAATTTTATAACAAACCTTGGAATCAATTTATTTGGAGAAGAGGTTCGTTGAAC
        1969
PF3D7_1148640,         GCACTTTAATTTTATAGCAAACCTTGGAATCAATTTATTTGGAGAAGAGGTTTGTTGAAC
        2200
PF3D7_1371300,         GCACTTTAATTTTATAGCAAACCTTGGAATCAATTTATTTGGAGAAGAGGTTTGTTGAAC
        2202
                       ********  ****** ******************** ************** *******

PF3D7_0532000,28S      TCAATTCAAAAAATTTCTTGAACGGGGTTT----------------------TCGGATTC
        1943
PF3D7_0726000,28S      TCAATTCAAAAAATTTCTTGAACGGGGTTT----------------------TCGGATTC
        1941
PF3D7_0112700,         TCAATTCAAAAAAAAAAAAAAAAAAAAATTAATTATAGAAGGAAAGGATTTTCGGATCC
        2029
PF3D7_1148640,         TCAATTCAAAAAATTTATGGCTGGGTTT------------------------TCGGATTC
        2236
PF3D7_1371300,         TCAATTCAAAAAATTTATGGCTGGGTTT------------------------TCGGATTC
        2238
                       *************                                       ****** *

PF3D7_0532000,28S      AGTTCA--------------------TTTTATTTTTTGTTTTGTAGCAATAGTAATTCG-
        1982
PF3D7_0726000,28S      AGTTCA--------------------TTTTATTTTTTGTTTTGTAGCAATAGTAATTCG-
        1980
PF3D7_0112700,         TTTGTACTTTTTTTTTTTTTTTTTTTTTTTTTTTTATGTTTTGTAGCAATAGTAATTCAT
        2089
PF3D7_1148640,         AGTTCA--------------------TTTTATTTTTTGTTTTGTAGCAATAGTAATTCG-
        2275
PF3D7_1371300,         AGTTCA--------------------TTTTATTTTTTGTTTTGTAGCAATAGTAATTCG-
        2277
                         *  *                    **** **** **********************

PF3D7_0532000,28S      TTTTTATGAATTATCCGAAGTGGTAAGGACTATCCTTGAAAAAAGGAGGGAACGGCTTTG
        2042
PF3D7_0726000,28S      TTTTTATGAATTATCCGAAGTGGTAAGGACTATCCTTGAAAAAAGGAGGGAACGGCTTTG
        2040
PF3D7_0112700,         TTCTTTTGAATTATCCATAGTGGTAAAAACTATCCATGAAACAAGGAGGGAACAGCAGCA
        2149
PF3D7_1148640,         TTTTTATGAATTATCCGAAGTGGTAAAAACTATCCTTGAAAAAAGGAGGGAACGGAGCAT
        2335
PF3D7_1371300,         TTTTTATGAATTATCCGAAGTGGTAAAAACTATCCTTGAAAAAAGGAGGGAACGGAGCAT
        2337
                       ** ** **********  ********  ******* ***** *********** *

PF3D7_0532000,28S      TAACTTG----------------------GTTTCTTCTGATTCCATTTTGCTTTGCTTAT
        2080
PF3D7_0726000,28S      TAACTTG----------------------GTTTCTTCTGATTCCATTTTGCTTTGCTTAT
        2078
PF3D7_0112700,         ATTATA-------------TAATTTTTGGGGTTCTTTGAATTCCTTATTATTTTTGCAAT
        2196
PF3D7_1148640,         ATACAGAAGGGAATTTAATTATTTTTTTTTTTTGAGTTACCTTCTTATGTGAAATACTCT
        2395
PF3D7_1371300,         ATACAGAAGGGAATTTAATTATTTTTTTTTTTTGAGTTACCTTCTTATGTGAAATACTCT
        2397
                                                      **        * * * *           *

PF3D7_0532000,28S      ACTCTTAATACTTGTATGAGCGTACCAACAACCGCATCAGGTCTCCAAGGTTAACAGCCT
        2140
PF3D7_0726000,28S      ACTCTTAATACTTGTATGAGCGTACCAACAACCGCATCAGGTCTCCAAGGTTAACAGCCT
        2138
```

```
PF3D7_0112700,         ATTCTTAATACTTGTACGAGCGTACCAATAACCGCATCAGGTCTCCAAGGTTAGTAGCCT
        2256
PF3D7_1148640,         TATATTCAATCAATAATGAGTGTACCAATAACCGCATCAGGTCTCCAAGGTTAGTAGCCT
        2455
PF3D7_1371300,         TATATTCAATCAATAATGAGTGTACCAATAACCGCATCAGGTCTCCAAGGTTAGTAGCCT
        2457
                         * ** *  *    * *** ******* ************************  *****

PF3D7_0532000,28S      CTGGTTAAATAGAAAAAAGTAGGTAAGGGAAGTCGGCAAAATAGATCCGTAACTTCGGGA
        2200
PF3D7_0726000,28S      CTGGTTAAATAGAAAAAAGTAGGTAAGGGAAGTCGGCAAAATAGATCCGTAACTTCGGGA
        2198
PF3D7_0112700,         CTGGTTAAATAGAAAAAAGTAGGTAAGGGAAGTCGGCAAAATAGATCCGTAACTTCGGGA
        2316
PF3D7_1148640,         CTGGTTAAATAGAAAAAAGTAAGTAAGGGAAGTCGGCAAAATAGATCCGTAACTTCGGGA
        2515
PF3D7_1371300,         CTGGTTAAATAGAAAAAAGTAAGTAAGGGAAGTCGGCAAAATAGATCCGTAACTTCGGGA
        2517
                       ********************* **************************************

PF3D7_0532000,28S      AAAGGATTGGCTCTGAGGACATTAGAAAAGAGAAGAAAAAAAGAGGGTCGAAAATAAAAT
        2260
PF3D7_0726000,28S      AAAGGATTGGCTCTGAGGACATTAGAAAAGAGAAGAAAAAAAGAGGGTCGAAAATAAAAT
        2258
PF3D7_0112700,         AAAGGATTGGCTCTGAGGACATTAGAAAAGAGAAGAAAAAAAGAGGGT-TGAGAATAAAA
        2375
PF3D7_1148640,         AAAGGATTGGCTCTGAGGACATTAGAAAAGAGAAGAAAAAAAAGGGGGTTGAAAATAAAA
        2575
PF3D7_1371300,         AAAGGATTGGCTCTGAGGACATTAGAAAAGAGAAGAAAAAAAAGGGGGTTGAAAATAAAA
        2577
                       ******************************************  ***    * *  ***

PF3D7_0532000,28S      TGCAGTCTTTATTTGCTTTTCTTT--CGATTTGCTTGTAATCTGCTTT-TTCT---TTTT
        2314
PF3D7_0726000,28S      TGCAGTCTTTATTTGCTTTTCTTT--CGATTTGCTTGTAATCTGCTTT-TTCT---TTTT
        2312
PF3D7_0112700,         TTGCAGATTTATTTGCTTTTCTCTCT-GATTTGCTTGTAAATTTTCTTTTTCTTTTTCTT
        2434
PF3D7_1148640,         TTACAGATTTATTTGCTTTATCTTTTCGATTTGGTTGTAAATTTGTTTTTCGTTTTTCTT
        2635
PF3D7_1371300,         TTACAGATTTATTTGCTTTATCTTTTCGATTTGGTTGTAAATTTGTTTTTCGTTTTTCTT
        2637
                       *      ************    *   ****** ******  *   ** *  *   * **

PF3D7_0532000,28S      CTTCTTTCTTTTTTTCTCCCCCTCTTTTCGCCTTCACTTTATTGTAATTTTATTACT---
        2371
PF3D7_0726000,28S      CTTCTTTCTTTTTTTCTCCCCCTCTTTTCGCCTTCACTTTATTGTAATTTTATTACT---
        2369
PF3D7_0112700,         CTTTTCTTTTTTTTTCTGTCCCTCTTTTCGTCTTCATTTTATTGTAATTTTTGTTACTTT
        2494
PF3D7_1148640,         C--TTTGTTTTTATTCTGTTCCTGTTTTCGCTTTCATTTTATTGTAATTTTAGTTACTTA
        2693
PF3D7_1371300,         C--TTTGTTTTTATTCTGTTCCTGTTTTCGCTTTCATTTTATTGTAATTTTAGTTACTTA
        2695
                       *   *   **** ****   *** ******  **** **************  *

PF3D7_0532000,28S      -TTAATTTGATACCTATAATGTTAACTCAGAACTGAAACGGACAAGGGGAATCCGACTGT
        2430
PF3D7_0726000,28S      -TTAATTTGATACCTATAATGTTAACTCAGAACTGAAACGGACAAGGGGAATCCGACTGT
        2428
PF3D7_0112700,         A-ATTTGATACATATATAATGTTAACTCAGAACTGAAACGGACAAGGGGAATCCGACTGT
        2553
```

```
PF3D7_1148640,           AATTTTTTGATATATATAATGTTAACTCAGAACTGAAACGGACAAGGGGAATCCGACTGT
        2753
PF3D7_1371300,           AATTTTTTGATATATATAATGTTAACTCAGAACTGAAACGGACAAGGGGAATCCGACTGT
        2755
                              *   * *  **********************************************

PF3D7_0532000,28S        TTAATTAAAACATAGCATTGTGAAAAGCCACAACTGGTATTAACACAATGTGATTTCTGC
        2490
PF3D7_0726000,28S        TTAATTAAAACATAGCATTGTGAAAAGCCACAACTGGTATTAACACAATGTGATTTCTGC
        2488
PF3D7_0112700,           TTAATTAAAACATAGCATTGTGAAAAGCCATAACTGGTATTAACACAATGTGATTTCTGC
        2613
PF3D7_1148640,           TTAATTAAAACATAGCATTGTGAAAAACCATAACTGGTATTAACACAATGTGATTTCTGC
        2813
PF3D7_1371300,           TTAATTAAAACATAGCATTGTGAAAAACCATAACTGGTATTAACACAATGTGATTTCTGC
        2815
                         ************************** *** *****************************

PF3D7_0532000,28S        CCAGTGCTTTGAATGTCAACTTGATGAA--ATTCAATCAAGCGCAGGTAAACGGCGGGAG
        2548
PF3D7_0726000,28S        CCAGTGCTTTGAATGTCAACTTGATGAA--ATTCAATCAAGCGCAGGTAAACGGCGGGAG
        2546
PF3D7_0112700,           CCAGTGCTTTGAATGTCAAATTGATGAA--ATTTAATTAAGCGCAGGTAAACGGCGGGAG
        2671
PF3D7_1148640,           CCAGTGCTTTGAATGTTAAGTTGATGAAAAATTCAATTAAGCGCAGGTAAACGGCGGGAG
        2873
PF3D7_1371300,           CCAGTGCTTTGAATGTTAAGTTGATGAAAAATTCAATTAAGCGCAGGTAAACGGCGGGAG
        2875
                         **************** ** ********  *** *** **********************

PF3D7_0532000,28S        TAACTATGACTCTCTTAAGGTAGCCAAATGCCTCGTCATCTAATTAGTGACGCGCATGAA
        2608
PF3D7_0726000,28S        TAACTATGACTCTCTTAAGGTAGCCAAATGCCTCGTCATCTAATTAGTGACGCGCATGAA
        2606
PF3D7_0112700,           TAACTATGACTCTCTTAAGGTAGCCAAATGCCTCGTCATCTAATTAGTGACGCGCATGAA
        2731
PF3D7_1148640,           TAACTATGACTCTCTTAAGGTAGCCAAATGCCTCGTCATCTAATTAGTGACGCGCATGAA
        2933
PF3D7_1371300,           TAACTATGACTCTCTTAAGGTAGCCAAATGCCTCGTCATCTAATTAGTGACGCGCATGAA
        2935
                         ************************************************************

PF3D7_0532000,28S        TGGATTAACGAGATTCCCACTGTCCCTACTTGCTATCTAGCGAAACCACAGCCAAGGGAA
        2668
PF3D7_0726000,28S        TGGATTAACGAGATTCCCACTGTCCCTACTTGCTATCTAGCGAAACCACAGCCAAGGGAA
        2666
PF3D7_0112700,           TGGATTAACGAGATTCCCACTGTCCCTACTTGCTATCTAGCGAAACCACAGCCAAGGGAA
        2791
PF3D7_1148640,           TGGATTAACGAGATTCCCACTGTCCCTACTTGCTATCTAGCGAAACCACAGCCAAGGGAA
        2993
PF3D7_1371300,           TGGATTAACGAGATTCCCACTGTCCCTACTTGCTATCTAGCGAAACCACAGCCAAGGGAA
        2995
                         ************************************************************

PF3D7_0532000,28S        CGGGCTTGGCAAAATCAGCGGGGAAAGAAGACCCTGTTGAGCTTTACTCTAGTCTGGCTT
        2728
PF3D7_0726000,28S        CGGGCTTGGCAAAATCAGCGGGGAAAGAAGACCCTGTTGAGCTTTACTCTAGTCTGGCTT
        2726
PF3D7_0112700,           CGGGCTTGGCAAAATCAGCGGGGAAAGAAGACCCTGTTGAGCTTTACTCTAGTCTGACTT
        2851
PF3D7_1148640,           CGGGCTTGGCAAAATCAGCGGGGAAAGAAGACCCTGTTGAGCTTTACTCTAGTCTGGCTT
        3053
```

```
PF3D7_1371300,          CGGGCTTGGCAAAATCAGCGGGGAAAGAAGACCCTGTTGAGCTTTACTCTAGTCTGGCTT
       3055
                        ******************************************************** ***

PF3D7_0532000,28S       TGTGAAACGACTTAAGAGGTGTAGCATAAGTGGGAGTAGAAACTGAA-------------
       2775
PF3D7_0726000,28S       TGTGAAACGACTTAAGAGGTGTAGCATAAGTGGGAGTAGAAACTGAA-------------
       2773
PF3D7_0112700,          TGTAAAACGACTTAAAAGGTGTAGATATAAGTGGGAGTAGGAAAATGAAA----------
       2901
PF3D7_1148640,          TGTGAAACGACTTAGAAGGTGTAGTATAAGTGGGAGTACAAAGAAGGGATCGAAAATTAA
       3113
PF3D7_1371300,          TGTGAAACGACTTAGAAGGTGTAGTATAAGTGGGAGTACAAAGAAGGGATCGAAAACTAA
       3115
                        *** **********  ********    *   **       *

PF3D7_0532000,28S       ----------ATATGTTTTTACGACAGTGAAATACCACTACTTTTAAAGTTGTTTTACTT
       2825
PF3D7_0726000,28S       ----------ATATGTTTTTACGACAGTGAAATACCACTACTTTTAAAGTTGTTTTACTT
       2823
PF3D7_0112700,          ----------TACGTTTTTTACAATAGTGAAATACCACTACTTTTAAAGTTGTTTTACTA
       2951
PF3D7_1148640,          TTCGATCTTTATGTTTTTGTACACAAATGAAATACCACTACTTTTACAGTTGTTTTACTT
       3173
PF3D7_1371300,          TTCGATCTTTATGTTTTTGTACACAAATGAAATACCACTACTTTTACAGTTGTTTTACTT
       3175
                                       *** ***   * ******************* ************

PF3D7_0532000,28S       ATCCATTTATAGGGAAATATATTATGCTTTATCCTTCGGGA-------------------
       2866
PF3D7_0726000,28S       ATCCATTTATAGGGAAATATATTATGCTTTATCCTTCGGGA-------------------
       2864
PF3D7_0112700,          ATCCATTGATAGGGATATATAAAACTTATAAATAATTTTTATTTTAAGTTACTTTTGAAT
       3011
PF3D7_1148640,          ATCTATTAATAGGGAAATATATTATGCTTTAATTCTTTGTG-------------------
       3214
PF3D7_1371300,          ATCTATTAATAGGGAAATATATTATGCTTTAATTCTTTGTG-------------------
       3216
                        *** *** ******* *****  *    * *    *

PF3D7_0532000,28S       -------------------------AGGCATTCTGATATTATTTGAATTTTTAAGAAACT
       2901
PF3D7_0726000,28S       -------------------------AGGCATTCTGATATTATTTGAATTTTTAAGAAACT
       2899
PF3D7_0112700,          TTAAGATATGTGTGCATTTATATCTATTAAATCCCATTTTGTATATATATATATATTATA
       3071
PF3D7_1148640,          --------------AA-------AAAGGCATTCTGATATTAT-TTGAATTTTTAAGAAAC
       3252
PF3D7_1371300,          --------------AA-------AAAGGCATTCTGATATTAT-TTGAATTTTTAAGAAAC
       3254
                                                 *   * **  ** ** * *  *  * *     *

PF3D7_0532000,28S       TGTTTCTTATCTTTCTCCCAATTTCTATGGAGACATAGCCAGGTGGGGAGTTTGACTGGG
       2961
PF3D7_0726000,28S       TGTTTCTTATCTTTCTCCCAATTTCTATGGAGACATAGCCAGGTGGGGAGTTTGACTGGG
       2959
PF3D7_0112700,          TATATGTGTATAATACGATTTTTTTTATGGAGACATAGTTAGGTGGGGAGTTTGACTGGG
       3131
PF3D7_1148640,          TTGTTTCTTATATTTCTCCCATTTCTATGGAGACATAGCCAGGTGGGGAGTTTGACTGGG
       3312
PF3D7_1371300,          TTGTTTCTTATATTTCTCCCATTTCTATGGAGACATAGCCAGGTGGGGAGTTTGACTGGG
       3314
                        *   *        *       *** *************  ********************
```

```
PF3D7_0532000,28S      GCGGTACATCTGTTAAATATTAACGCAGATGTCCAAAGACAAGCTCAAAGAGAACAGAAA
      3021
PF3D7_0726000,28S      GCGGTACATCTGTTAAATATTAACGCAGATGTCCAAAGACAAGCTCAAAGAGAACAGAAA
      3019
PF3D7_0112700,         GCGGTACATCTGTTAAAAAATAACGCAGATGTCCAAAGACAAGCTCAAAGAGAACAGAAA
      3191
PF3D7_1148640,         GCGGTACATCTGTTAAAAAATAACGCAGATGTCCAAAGACAAGCTCAATGAGAACAGAAA
      3372
PF3D7_1371300,         GCGGTACATCTGTTAAAAAATAACGCAGATGTCCAAAGACAAGCTCAATGAGAACAGAAA
      3374
                       ***************** * **************************** ***********

PF3D7_0532000,28S      TCTCTTGTAGACTAAAAGGGGAAAAGCTTGTTTGATTTCTATTTTCAGAACAAGTAGAAA
      3081
PF3D7_0726000,28S      TCTCTTGTAGACTAAAAGGGGAAAAGCTTGTTTGATTTCTATTTTCAGAACAAGTAGAAA
      3079
PF3D7_0112700,         TCTCTTGTAGACTAAAAGGGGAAAAGCTTGTTTGATTTTTACTTTCAGAATGAGTAGAAA
      3251
PF3D7_1148640,         TCTCGTGTAGACTAAAAGGGGAAAAGCTTGTTTGATTTCTATTTTCAGAAAGAATAGAAA
      3432
PF3D7_1371300,         TCTCGTGTAGACTAAAAGGGGAAAAGCTTGTTTGATTTCTATTTTCAGAAAGAATAGAAA
      3434
                       **** ********************************* ** ********  * ******

PF3D7_0532000,28S      ACGTGAAAGCGTGGCCTATCGATCCTTTATATTTGCAAAATGACGTAAAT---TACTTAC
      3138
PF3D7_0726000,28S      ACGTGAAAGCGTGGCCTATCGATCCTTTATATTTGCAAAATGACGTAAAT---TACTTAC
      3136
PF3D7_0112700,         ACGTGAAAGCGTGGCCTATCGATCCTTTATATTTGCAAAATGACGTAATAAATTACTTAC
      3311
PF3D7_1148640,         ACGTGAAAGCGTGGCCTATCGATCCTTTATTTTTGCAAAATGTATTAATAACA---TTCT
      3489
PF3D7_1371300,         ACGTGAAAGCGTGGCCTATCGATCCTTTATTTTTGCAAAATGTATTAATAACA---TTCT
      3491
                       ****************************** ***********   ***        **

PF3D7_0532000,28S      TACTGTGCATATAGAGGTGTCTGAAAAGTTACCACAGGGATAACTGGCTTGTGGCTGCCA
      3198
PF3D7_0726000,28S      TACTGTGCATATAGAGGTGTCTGAAAAGTTACCACAGGGATAACTGGCTTGTGGCTGCCA
      3196
PF3D7_0112700,         TACTGTGCATATAGAGGTGTCTGAAAAGTTACCACAGGGATAACTGGCTTGTGGCTGCCA
      3371
PF3D7_1148640,         GACTGTGCATATAAAGGTGTCTGAAAAGTTACCACAGGGATAACTGGCTTGTGGCTGCCA
      3549
PF3D7_1371300,         GACTGTGCATATAAAGGTGTCTGAAAAGTTACCACAGGGATAACTGGCTTGTGGCTGCCA
      3551
                        ************ **********************************************

PF3D7_0532000,28S      AGCGCTCCTAGCGACGTAGCTTTTTGATCCTTCGATGTCGGCTCTTCCTATCATTGGGAC
      3258
PF3D7_0726000,28S      AGCGCTCCTAGCGACGTAGCTTTTTGATCCTTCGATGTCGGCTCTTCCTATCATTGGGAC
      3256
PF3D7_0112700,         AGCGCTCTTAGCGACGTAGCTTTTTGATCCTTCGATGTCGGCTCTTCCTATCATTGGGAC
      3431
PF3D7_1148640,         AGCGCTCTTAGCGACGTAGCTTTTTGATCCTTCGATGTCGGCTCTTCCTATCATTGGGAC
      3609
PF3D7_1371300,         AGCGCTCTTAGCGACGTAGCTTTTTGATCCTTCGATGTCGGCTCTTCCTATCATTGGGAC
      3611
                       ******* ****************************************************

PF3D7_0532000,28S      GCAGAAGTCTCAAAGTGTCGGATTGTTCACCCGCTAATAGGGAACGTGAGCTGGGTTTAG
      3318
```

```
PF3D7_0726000,28S       GCAGAAGTCTCAAAGTGTCGGATTGTTCACCCGCTAATAGGGAACGTGAGCTGGGTTTAG
        3316
PF3D7_0112700,          GCAGAAGTCTCAAAGTGTCGGATTGTTCACCCGCTAATAGGGAACGTGAGCTGGGTTTAG
        3491
PF3D7_1148640,          GCAGAAGTCTCAAAGTGTCGGATTGTTCACCCGCTAATAGGGAACGTGAGCTGGGTTTAG
        3669
PF3D7_1371300,          GCAGAAGTCTCAAAGTGTCGGATTGTTCACCCGCTAATAGGGAACGTGAGCTGGGTTTAG
        3671
                        ************************************************************

PF3D7_0532000,28S       ACCGTCGTGAGACAGGTTAGTTTTACCCTACTGATGAATTTTATTATATTTTTTA-----
        3373
PF3D7_0726000,28S       ACCGTCGTGAGACAGGTTAGTTTTACCCTACTGATGAATTTTATTATATTTTTTA-----
        3371
PF3D7_0112700,          ACCGTCGTGAGACAGGTTAGTTTTACCCTACTGATGAATAATATTATTATATTTTTT--A
        3549
PF3D7_1148640,          ACCGTCGTGAGACAGGTTAGTTTTACCCTACTGATGAATTTTATTATATTTTTTTATATAT
        3729
PF3D7_1371300,          ACCGTCGTGAGACAGGTTAGTTTTACCCTACTGATGAATTTTATTATATTTTTTTATATAT
        3731
                        ***************************************  ******  * ***

PF3D7_0532000,28S       -TATATACATATAGTATTGTGACAGTAATCCAACTTGGTACGAGAGGATTAGTTGGTTCA
        3432
PF3D7_0726000,28S       -TATATACATATAGTATTGTGACAGTAATCCAACTTGGTACGAGAGGATTAGTTGGTTCA
        3430
PF3D7_0112700,          TATACAATATATAGTATTGTGACAGTAATCCAACTTGGTACGAGAGGATTAGTTGGTTCA
        3609
PF3D7_1148640,          ATATATACATATAATATTGTGACAGTAATCCAACTTGGTACGAGAGGATTAGTTGGTTCA
        3789
PF3D7_1371300,          ATATATACATATAGTATTGTGACAGTAATCCAACTTGGTACGAGAGGATTAGTTGGTTCA
        3791
                              * ***** **********************************************

PF3D7_0532000,28S       GACAATTGGTACAGCAATTGGTTGACAAACCAGTGTTGCGAAGCTAAGTCTGTTGGATAA
        3492
PF3D7_0726000,28S       GACAATTGGTACAGCAATTGGTTGACAAACCAGTGTTGCGAAGCTAAGTCTGTTGGATAA
        3490
PF3D7_0112700,          GACAATTGGTACAGCAATTGGTTGATAAACCAGTGTTGCGAAGCTAAGTCTGTTGGATAA
        3669
PF3D7_1148640,          GACAATTGGTACAGCAATTGGTTGATAAGCCAGTGTTGCGAAGCTAAGTCTGTTGGATAA
        3849
PF3D7_1371300,          GACAATTGGTACAGCAATTGGTTGATAAGCCAGTGTTGCGAAGCTAAGTCTGTTGGATAA
        3851
                        ************************* ** *******************************

PF3D7_0532000,28S       TGGCTGAACGCCTCTTAAGCCAGAAACCATGCTGATTAAACAATACTATTTCGTTCTTTT
        3552
PF3D7_0726000,28S       TGGCTGAACGCCTCTTAAGCCAGAAACCATGCTGATTAAACAATACTATTTCGTTCTTTT
        3550
PF3D7_0112700,          TGGCTGAACGCCTCTTAAGCCAGAACCCATGCTGATTAGACAATTCTAAATTTGATCTTT
        3729
PF3D7_1148640,          TGACTGAACGCCTCTTAAGTCAGAACCCATACTGATTAGACAATTTTATATTGTTCTTTT
        3909
PF3D7_1371300,          TGACTGAACGCCTCTTAAGTCAGAACCCATACTGATTAGACAATTTTATATTGTACTTTT
        3911
                        ** **************** ***** **** ******* *****  **  *      ***

PF3D7_0532000,28S       TTGTTTCTTTTTTTACTGC-----------------------------TAATGTAAAAGA
        3583
PF3D7_0726000,28S       TTGTTTCCTTTTTTACTGC-----------------------------TAATGTAAAAGA
        3581
```

```
PF3D7_0112700,            TTGTTAAACGATAAAAATTATATATATATTTTTTTTATCTTTACTGCATAATGTAAAAGA
       3789
PF3D7_1148640,            TGTTTAAATATAAAAATATTATTA----------TTTTTTTTTACTGCTAATGTAAATGA
       3959
PF3D7_1371300,            TGTTTAAATATAAAAATATTATTA----------TTTTTTTTTACTGCTAATGTAAATGA
       3961
                          *  **         *                                 ********* **

PF3D7_0532000,28S         GAAATTATATATGTACCTTTTTTTGGTACATA-------AATAATCCTATCACCTTTT--
       3634
PF3D7_0726000,28S         GAAATTATATATGTACCTTTTTTTGGTACATA-------AATAATCCTATCACCTTTT--
       3632
PF3D7_0112700,            GAAATTATACATATATATATATATATATATTAGAAAAGAAAAAATGATGTAATTAATTAT
       3849
PF3D7_1148640,            GAAATTATACATATATTAGAAAAAA--------------------AATGTAATTAATTGT
       3999
PF3D7_1371300,            GAAATTATACATATATTAGAAAAAA--------------------AATGTAATTAATTGT
       4001
                          ********* ** **                                * * *    **

PF3D7_0532000,28S         TC--GTGAGCTGTGAGCTGTGAGCTAGGCAATA---------------------------
       3665
PF3D7_0726000,28S         TC--GTGAGCTGTGAGCTGTGAGCTAGGCAATA---------------------------
       3663
PF3D7_0112700,            TATTATTATTTTTTTTTTTTTTTAGTATTAATATATAATTATGAATATATAAATAATCCT
       3909
PF3D7_1148640,            TT-------------TTTTTTCGCTTTATAA--------------TATATCAATAATCCT
       4032
PF3D7_1371300,            TT-------------TTTTTTCGCTTTATAA--------------TATATCAATAATCCT
       4034
                          *                * *         **

PF3D7_0532000,28S         ------------------------------------------------------------
       3665
PF3D7_0726000,28S         ------------------------------------------------------------
       3663
PF3D7_0112700,            ATATCTTTATATAAAAATTAAATTTTTAATTTTATTAGAATTTTTTTTTTTTATATATAT
       3969
PF3D7_1148640,            ATCTTTTTATATATATGTATGT--------GTATACATATATATATATGTATATATAAAT
       4084
PF3D7_1371300,            ATCTTTTTATATATATGTATGT--------GTATACATATATATATATGTATATATAAAT
       4086


PF3D7_0532000,28S         -----GTTC-TTTAACAACAAAAAAGAATTGCACAAACTGTAGACGACTTTTTTGCCTCA
       3719
PF3D7_0726000,28S         -----GTTC-TTTAACAACAAAAAAGAATTGCACAAACTGTAGACGACTTTTTTGCCTCA
       3717
PF3D7_0112700,            AGATTGTAATTTAACAACAAAAAGTATTAAGCCCAAATCGTAGACGACTTTTCTGTCTCA
       4029
PF3D7_1148640,            AGATTGTAATTTAACAACAAAAAGTGT--TAAGCATATTGTAGACGACTTTTCAGTCTCA
       4142
PF3D7_1371300,            AGATTGTAATTTAACAACAAAAAGTGT--TAAGCATATTGTAGACGACTTTTCAGTCTCA
       4144
                               **   ** *  *  ****          ** *  *************  * ****

PF3D7_0532000,28S         GGGTGCTGTAAACATGAAAGTAAACTTTGTTTTACGATCTGTTGAGGCTTATCCCTAGTG
       3779
PF3D7_0726000,28S         GGGTGCTGTAAACATGAAAGTAAACTTTGTTTTACGATCTGTTGAGGCTTATCCCTAGTG
       3777
PF3D7_0112700,            GAGTACTGTAAACATGAGAGTAAACTTTGTTTTACGATCTGTTGAGGTTTATCTCTTGTG
       4089
```

```
PF3D7_1148640,          GAGTGCTGTAAACATGAGAGTAAACTAAGTTTTACGATCTGTTGAGGCTTATCTCTTGTG
        4202
PF3D7_1371300,          GAGTGCTGTAAACATGAGAGTAAACTAAGTTTTACGATCTGTTGAGGCTTATCTCTTGTG
        4204
                        * ** ************ ********  ****************** ***** ** ***

PF3D7_0532000,28S       GTAAAGTGTTT----     3790
PF3D7_0726000,28S       GTAAAGTGTTT----     3788
PF3D7_0112700,          ACATTGAGCTAATTT     4104
PF3D7_1148640,          ACA------------     4205
PF3D7_1371300,          ACAATTAGCTA----     4215
                          *
```
