# Supplementary material for: Detection of Developmental Asexual Stage-Specific RNA Editing Events in Plasmodium falciparum 3D7 Malaria Parasite
Source: Microorganisms. 2024 Jan 10;12(1):137. doi: 10.3390/microorganisms12010137 (PMC10819399; doi:10.3390/microorganisms12010137)
Supplement: Supplementary file 1 [file microorganisms-12-00137-s001.zip › Supplementary File S2.pdf]

5 amino acids change in PF3D7_0216900 due to RNA editing

```
CLUSTAL O(1.2.4) multiple sequence alignment


Unedited      MEEKKKINNKSNSRVSNDDTNKEKRKKLKPIQVRRSIKDIIISHNPYDYIYNYKGNDIDI   60
Edited        MEEKKKINNKSNSRVSNDDTNKEKRKKLKPIQVRRSIKDIIISHNPYDYIYNYKGNDIDI   60
              ************************************************************

Unedited      FDINKHDKIVKDRTSEIEENSNIFIENEILDNNEMLLRKELNELINKDDLSEDMKNDIKA   120
Edited        FDINKHDKIVKDRTSEIEENSNIFIENEILDNNEMLLRKELSELINKDDLSEDMKNDIKA   120
              *****************************************.******************

Unedited      LYIEVQEMYLILKNDIKNNIPSSDEIIKLYLADDQKDKSTNIIWKRFCFYKLLSDKLNDL   180
Edited        LYIEVQEMYLILKNDIKNNIPSSDEIIKLYLADDQKDKSTNMIWKRICFYKLLSDKLNDL   180
              *****************************************:****:*************

Unedited      HISTISSYRHTYLKTIYIWYKKNKKLLFNTDDNKEVFGGNNIVGEINEVDEKNESDEKNE   240
Edited        HISTISSYRHTCLKTIYIWYKKNKKLLFNTDDNKEVFGGNNIVGEINEVDEKNESDEKNE   240
              *********** ************************************************

Unedited      VDEKNEGGEKNVDEKNEGGEKNVDEKNEGDEKNITNQNEIIKNKDPLNCHTKKEETEKEM   300
Edited        VDEKNEGGEKNVDEKNEVGEKNVDEKNEGDEKNITNQNEIIKNKDPLNCHTKKEETEKEM   300
              ***************** ******************************************

Unedited      KKDYAKKISHNFDETLQEEMNKIKKEHEIKENDINLLVYNEEPHDVLNKYTFPNDVFLLN   360
Edited        KKDYAKKISHNFDETLQEEMNKIKKEHEIKENDINLLVYNEEPHDVLNKYTFPNDVFLLN   360
              ************************************************************

Unedited      NTKISDKNIKNVKEEQNVVSNDLNVLLLRNDKDEEDKYAKGICEHVSLDIFINNNDAFNI   420
Edited        NTKISDKNIKNVKEEQNVVSNDLNVLLLRNDKDEEDKYAKGICEHVSLDIFINNNDAFNI   420
              ************************************************************

Unedited      NTNDAFNINTNDTFNINTNDAFNINTNDAFNINTNDAFNINTNDTFNINTNDTFNIKTND   480
Edited        NTNDAFNINTNDTFNINTNDAFNINTNDAFNINTNDAFNINTNDTFNINTNDTFNIKTND   480
              ************************************************************

Unedited      TLSINNYNLDIKEEHKNVPIPLHTNKIKELEEEIKKQKLLIKKKEIEIINSPIGIKFKDI   540
Edited        TLSINNYNLDIKEEHKNVPIPLHTNKIKELEEEIKKQKLLIKKKEIEIINSPIGIKFKDI   540
              ************************************************************

Unedited      FGKFQDINN   549
Edited        FGKFQDINN   549
              *********
```
