# Supplementary material for: Detection of Developmental Asexual Stage-Specific RNA Editing Events in Plasmodium falciparum 3D7 Malaria Parasite
Source: Microorganisms. 2024 Jan 10;12(1):137. doi: 10.3390/microorganisms12010137 (PMC10819399; doi:10.3390/microorganisms12010137)
Supplement: Supplementary file 1 [file microorganisms-12-00137-s001.zip › Supplementary Table S1.pdf]

Supplementary Table S1. Selected genes for our RNA editing study in *Plasmodium falciparum* 3D7

Genes ID	Genes Name	Oligonucleotides sequences	Product size
PF3D7_0532000	28S rRNA		
PF3D7_0112700	28S rRNA	3D7700 F 596; ACAAGGGGAATCCGACTGTT<br>3D7700 R 596; CCAGTCAAACTCCCCACCT	
PF3D7_1148640	28S rRNA		
PF3D7_1371300	28S rRNA		
PF3D7_0801100	28S rRNA		
PF3D7_0216900	Protein of unknown function	CPP 900F; CAACAAGTCAAACAGTCGAGTTTC<br>CPP 900 R; CCCCCAAACACTTCTTTGTT	600 bp
PF3D7_0501200	Parasite-infected erythrocyte surface protein	PIPES FW; TTAATTTTTAGGAACATTTTTGGAA<br>PIPES Rev; TTCATCATGATTTGGCGTGT	954 bp
PF3D7_1227200,	potassium channel K1	Kchanl1Fw; TCTGATGGAGGAAGAAAACGA<br>Kchanl1Rev; GCAGAATCTTTCATCATCAAATCA	557 bp<br>ND
PF3D7_1149000;	antigen 332, DBL-like protein	FP1 antigen 332; GGAGACCGAACAAACAGAGG<br><br>RP1 antigen 332;<br>TGTTCTTCCGGAATATCATCGGT	750bp<br>ND
PF3D7_0815200	importin subunit beta	Imp B fw; GGACCTTATCTATGGCATCAGC<br>Imp B Re; CAGGCAGCTGTATCACGAAC	260 bp<br>ND

ND= RNA editing not detected
